# RNA helicase DDX6 governs ASC speck formation in P-bodies and the transition to stress granules via phase separation during inflammasome activation

**DOI:** 10.1038/s41421-026-00898-1

**Published:** 2026-06-24

**Authors:** Rudi Mao, Yingqiao Liu, Zhenyu Fan, Yanfeng Li, Luyu Yang, Qingqing Xie, Hong-Peng Dong, Mingjun Bi, Chengjiang Gao, Zhe Yang, Tao Xu, Xiaopeng Qi

**Affiliations:** 1 Key Laboratory for Experimental Teratology of the Ministry of Education, Advanced Medical Research Institute, Qilu Hospital, Cheeloo College of Medicine, Shandong University, Jinan, Shandong China; 2 Department of Radiation Oncology, Shandong Provincial Hospital Affiliated to Shandong First Medical University, Jinan, Shandong China; 3 Department of Immunology and Key Laboratory of Infection and Immunity of Shandong Province & Key Laboratory for Experimental Teratology of Ministry of Education, Department of Immunology, School of Basic Medical Sciences, Shandong University, Jinan, Shandong China; 4 State Key Laboratory for Innovation and Transformation of Luobing Theory; Key Laboratory of Cardiovascular Remodeling and Function Research of MOE, NHC, CAMS and Shandong Province; Department of Cardiology, Qilu Hospital of Shandong University, Jinan, Shandong China

**Keywords:** Innate immunity, Cell death

## Abstract

The recruitment and condensation of apoptosis-associated speck-like protein containing a CARD (ASC) are critical for ASC speck formation and inflammasome activation. However, how this process occurs efficiently in vivo remains unclear. Here, we identified the RNA helicase DDX6 as an ASC-interacting protein through immunoprecipitation‒mass spectrometry (IP‒MS) analysis. DDX6 promotes the activation of both NLRP3 and AIM2 inflammasomes by facilitating the recruitment of ASC to these receptors through its RNA helicase activity. Mechanistically, DDX6 functions as a scaffold protein for processing body (P-body) assembly and drives ASC speck formation in P-bodies via liquid‒liquid phase separation (LLPS). We report that membrane integrity is associated with stress granule (SG) formation and that in *Caspase-1*^*–/–*^, *Gsdmd*^*–/–*^, or NINJ1-inhibited cells, DDX6 mediates initial ASC speck assembly in P-bodies, followed by their transition to SGs during inflammasome activation. DDX6 deficiency in macrophages increases host susceptibility to *Listeria* infection. Our results establish that DDX6 orchestrates ASC recruitment, speck formation, and subsequent transition through LLPS-mediated mechanisms, offering new insights into inflammasome assembly and potential therapeutic approaches for inflammasome-related diseases.

## Introduction

Inflammasomes are supramolecular complexes in the cytoplasm that serve as critical regulators of the innate immune system in response to pathogen- and damage-associated stimuli^1^. Inflammasome activation is tightly regulated, as uncontrolled activation can lead to various rare genetic syndromes, including autoinflammatory, neurodegenerative, infectious, and cardiovascular diseases^2^. The assembly of canonical inflammasomes, such as the NLRP3 and AIM2 inflammasomes, begins with the oligomerization of sensor proteins (NLRP3 or AIM2), followed by the sequential recruitment of the adaptor protein ASC and the effector protein caspase-1^3,4^. Cryo-electron microscopy (cryo-EM) structural analyses have revealed that disc-shaped active NLRP3 oligomers expose PYD filaments, leading to the formation of NLRP3^PYD^ nucleation seeds. These seeds recruit ASC, facilitating PYD filament elongation and ASC speck formation^5,6^. However, the in vivo transition from NLRP3 nucleation seeds to ASC filament elongation remains unclear.

Protein phase separation, or biomolecular condensation, is a fundamental physical process underlying the formation of membraneless organelles via the supramolecular assembly of proteins, nucleic acids, and other biomolecules^7,8^. P-bodies (PBs) and stress granules (SGs) are dynamic intracellular condensates formed through phase separation of proteins and RNAs, which mediate mRNA translational repression^9–12^. DEAD/H-box RNA helicases are ATP-dependent RNA-binding proteins that exhibit highly cooperative binding to ATP and RNA. Among these proteins, DDX6 is a key regulator of P-body assembly and stress granule biogenesis under multiple conditions^13–15^. In addition to its structural role, DDX6 also modulates cellular plasticity by suppressing the translation of target mRNAs and maintaining P-body homeostasis^16^. During inflammasome activation, DDX3X and DHX33 were identified as NLRP3-binding proteins that link cytosolic RNA sensing to NLRP3 inflammasome assembly^17,18^. Although RNA and RNA helicases are essential for the activation of multiple inflammasomes^19–21^, the specific contributions of P-bodies and their scaffold proteins — particularly RNA helicase-mediated phase separation — to ASC speck formation and subsequent inflammasome activation remain poorly understood.

Here, we identify DDX6 as an ASC-interacting protein that binds to the PYD domain of ASC, facilitating the recruitment of ASC alongside the inflammasome receptors NLRP3 and AIM2. DDX6 deficiency impairs NLRP3 and AIM2 inflammasome activation, compromising host defense against *Listeria* infection. Mechanistically, DDX6 acts as a scaffold protein for P-body assembly, promoting ASC recruitment to nucleation seeds and speck formation via liquid‒liquid phase separation (LLPS) during inflammasome activation. The ATPase activity of DDX6 is essential for this phase separation, P-body formation, and subsequent ASC speck assembly. Intriguingly, in cells lacking caspase-1, GSDMD, or NINJ1, DDX6 mediates ASC speck translocation to SGs upon inflammasome activation, with DDX6–ASC localizing to P-bodies and SGs in a mutually exclusive manner. Together, these findings demonstrate that DDX6 orchestrates ASC speck formation and transition through LLPS-driven condensation, underscoring its pivotal role in inflammasome regulation.

## Results

### DDX6 interacts with ASC

**Fig. 1:**
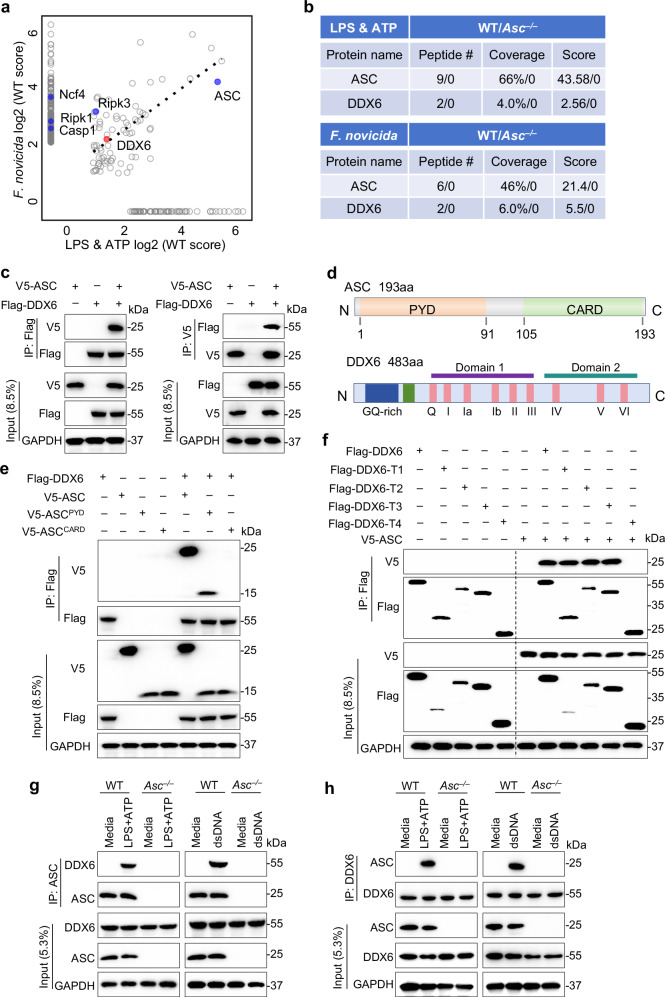
Characterization of ASC-interacting protein DDX6. **a** Results from the mass spectrometry analysis of the IP product with ASC antibody from WT and *Asc*^*–/–*^ BMDMs treated with LPS plus ATP (LPS, 500 ng/mL for 4 h and ATP, 5 mM for 20 min) for NLRP3 inflammasome activation and infected with *F. novicida* (MOI = 100, 12 h) for AIM2 inflammasome activation. **b** Detected peptides of ASC and DDX6 from WT and *Asc*^*–/–*^ BMDMs by IP-MS in **a**. **c** Immunoblot analysis of FLAG-DDX6 co-IP with V5-ASC and reciprocal V5-ASC co-IP with FLAG-DDX6 from the lysates of HEK293T cells transfected with the indicated plasmids. **d** Domain architecture of full-length ASC and DDX6 proteins. **e** Immunoblot analysis of FLAG-DDX6 co-IP with V5-ASC, V5-ASC^PYD^, and V5-ASC^CARD^ from the lysates of HEK293T cells transfected with the indicated plasmids. **f** Results from the immunoblot analysis of full-length FLAG-DDX6 and truncated FLAG-DDX6-T1/T2/T3/T4 co-IP with V5-ASC from the lysates of HEK293T cells transfected with the indicated plasmids. **g** Co-IP analysis of endogenous ASC interactions with DDX6 in WT and *Asc*^*–/–*^ BMDMs treated with the NLRP3 activator LPS plus ATP (LPS, 500 ng/mL for 4 h and ATP, 5 mM for 20 min) or transfected with the AIM2 activator dsDNA (1.5 μg, 1 h). **h** Co-IP analysis of endogenous DDX6 interaction with ASC in WT and *Asc*^*–/–*^ BMDMs treated with the NLRP3 activator LPS plus ATP (LPS, 500 ng/mL for 4 h and ATP, 5 mM for 20 min) or transfected with the AIM2 activator dsDNA (1.5 μg, 1 h). Data are from 2 (**a**, **b**) or are representative of 3 independent experiments with similar results (**c**, **e**–**h**).

The transition from NLRP3 or AIM2 nucleation seeds to ASC filament elongation is a critical step in ASC speck formation during inflammasome activation^4,5^. To investigate the regulatory mechanism by which intracellular ASC is incorporated into nucleation seeds for filament growth and speck formation in vivo, we performed ASC immunoprecipitation‒mass spectrometry (IP‒MS) analysis in wild-type (WT) and *Asc*^*–/–*^ bone marrow-derived macrophages (BMDMs). Cells were stimulated with LPS plus ATP (NLRP3 inflammasome activation) or infected with *Francisella novicida* (AIM2 inflammasome activation), as previously described^22^. Among the top ASC-interacting proteins identified, the RNA helicase DDX6 emerged as a key candidate due to its established role as a global regulator of phase-separated P-body assembly (Fig. 1a, b; Supplementary Table S1)^23,24^. Co-immunoprecipitation (co-IP) assays confirmed the interaction between ASC and DDX6 (Fig. 1c). Further mapping revealed that the PYD domain of ASC and Domain 1 of DDX6 mediate this interaction (Fig. 1d–f; Supplementary Fig. S1a). In WT BMDMs, endogenous co-IP assays with anti-ASC and anti-DDX6 antibodies demonstrated that ASC and DDX6 interact following NLRP3 (LPS plus ATP) or AIM2 (dsDNA transfection) inflammasome activation (Fig. 1g, h).

To evaluate the association between DDX6 expression and infectious disease status, we analyzed RNA sequencing source data from peripheral blood mononuclear cells (PBMCs) and monocytes from 284 healthy donors and 125 patients with sepsis (GSE205672)^25^. Notably, DDX6 expression was significantly higher in both PBMCs and monocytes from healthy individuals than in those from patients with sepsis (Supplementary Fig. S1b). To examine cell type-specific DDX6 expression patterns during disease progression, we analyzed a published single-cell RNA sequencing (scRNA-seq) dataset from the bronchoalveolar lavage fluid (BALF) of patients with COVID-19 with mild/moderate (*n* = 3) or severe/critical (*n* = 4) symptoms (GSE158055; Supplementary Fig. S1c), comprising a total of 11,482 cells^26^. Following batch effect correction (Supplementary Fig. S1d), we identified six major cell populations (myeloid cells, NK cells, T cells, epithelial cells, B cells, and plasma cells) across 15 distinct clusters based on marker gene expression (Supplementary Fig. S1e, f). Notably, compared with that in mild/moderate cases, DDX6 expression was most significantly reduced in myeloid and NK cells but not in T cells from severe/critical cases (Supplementary Fig. S1g). These findings suggest that DDX6, an ASC-interacting protein, may function as a positive regulator of host defense against pathogenic infections.

### DDX6 promotes NLRP3 and AIM2 inflammasome activation

**Fig. 2:**
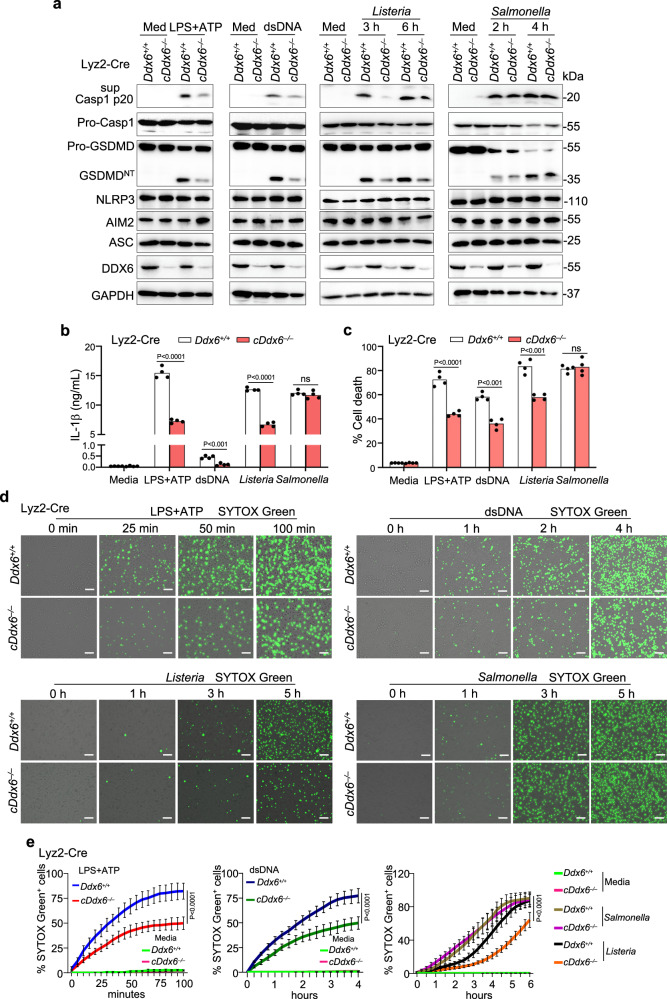
DDX6 promotes both NLRP3 and AIM2 inflammasome activation. **a** Immunoblot analysis of pro-caspase-1 (Pro-Casp1), its subunit p20, DDX6, NLRP3, AIM2, ASC, and full-length and cleaved GSDMD (GSDMD^NT^) in Lyz2-Cre-expressing *Ddx6*^*fl/fl*^ (*cDdx6*^*–/–*^) and *Ddx6*^*+/+*^ BMDMs without treatment (Med) or stimulated with LPS (500 ng/mL, 4 h) and ATP (5 mM, 60 min) for NLRP3 inflammasome activation; transfected with dsDNA (1.5 μg, 2 h) for AIM2 inflammasome activation; and infected with *Listeria monocytogenes* (MOI = 50, 3 h and 6 h) for both NLRP3 and AIM2 inflammasome activation and Salmonella enterica Typhimurium (MOI = 3, 2 h and 4 h) for NLRC4 inflammasome activation. **b**, **c** IL-1β release (**b**) and LDH secretion (**c**) in WT and *Ddx6*-deficient BMDMs without treatment (Media) or stimulated with LPS (500 ng/mL, 4 h) and ATP (5 mM, 60 min), transfected with dsDNA (1.5 μg, 2 h), infected with *Listeria monocytogenes* (MOI = 50, 6 h), and infected with *Salmonella enterica* Typhimurium (MOI = 3, 4 h) (*n* = 4 biologically independent samples). **d** Representative images of SYTOX Green-positive cells among WT and *Ddx6*-deficient BMDMs stimulated with LPS (500 ng/mL) and ATP (5 mM), transfected with dsDNA (1.5 μg), and infected with *Listeria monocytogenes* (MOI = 50) and *Salmonella enterica* Typhimurium (MOI = 3) for the indicated times. Scale bars: 50 μm. **e** Real-time live-cell imaging and death analysis of WT and *Ddx6*-deficient BMDMs without treatment (Media) or stimulated with LPS (500 ng/mL) and ATP (5 mM), transfected with dsDNA (1.5 μg), and infected with *Listeria monocytogenes* (MOI = 50) and *Salmonella enterica* Typhimurium (MOI = 3) for the indicated times (*n* = 8 random fields; 3 independent experiments). Data are from 3 (**b**, **c**) or are representative of 3 independent experiments with similar results (**a**, **d**, **e**). Data represent the mean ± SEM for **b**, **c**, 2-sided Student’s *t*-test without multiple-comparisons correction, two-way ANOVA for **e**. *P* values area indicated in the graphs.

To investigate the role of DDX6 in inflammasome regulation, we generated myeloid-specific DDX6-knockout mice by crossing *Ddx6*^*fl/fl*^ mice with Lyz2-Cre mice (Supplementary Fig. S2a, b). Flow cytometry revealed equivalent frequencies of CD11b^+^F4/80^+^ BMDMs and CD11c^+^F4/80^+^ alveolar macrophages in Lyz2-Cre^+^ *Ddx6*^*fl/fl*^ (*cDdx6*^*–/–*^) and *Ddx6*^*+/+*^ mice (Supplementary Fig. S2c), indicating that DDX6 loss does not compromise macrophage development. We stimulated BMDMs from Lyz2-Cre^+^ *Ddx6*^*fl/fl*^ and *Ddx6*^*+/+*^ mice with canonical NLRP3 and AIM2 agonists and the relevant pathogens. Cleaved caspase-1, mature GSDMD-N (Fig. 2a; Supplementary Fig. S2d), inflammasome-dependent release of the cytokine IL-1β (Fig. 2b), and leakage of the membrane rupture marker lactate dehydrogenase (LDH) (Fig. 2c) were markedly decreased in *Ddx6*-deficient cells after treatment with LPS plus ATP (NLRP3), transfection with dsDNA (AIM2), or infection with *Francisella novicida* (AIM2) or *Listeria monocytogenes* (NLRP3 and AIM2). Real-time cell-death assays confirmed that DDX6 loss protected against pyroptosis under these same conditions (Fig. 2d, e). In contrast, the production of the inflammasome-independent cytokines IL-6 and TNF remained unchanged (Supplementary Fig. S2e), and activation of the NLRC4 inflammasome by *Salmonella enterica* Typhimurium was unaffected by DDX6 deficiency (Fig. 2a–e; Supplementary Fig. S2e). Thus, DDX6 selectively facilitates NLRP3- and AIM2-mediated inflammasome activation and subsequent pyroptosis.

### DDX6 drives ASC recruitment to NLRP3 and AIM2 via its ATPase activity

**Fig. 3:**
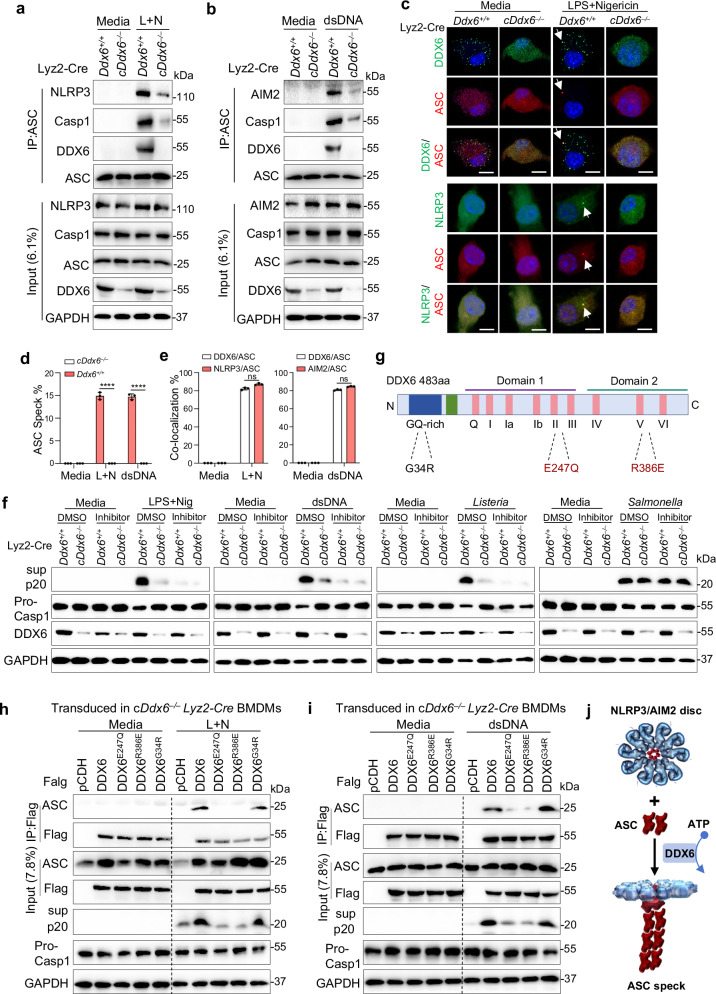
DDX6 mediates the interaction of ASC with NLRP3 and AIM2 via its ATPase activity. **a** Co-IP analysis of endogenous ASC interactions with NLRP3, caspase-1, and DDX6 in Lyz2-Cre-expressing *Ddx6*^*fl/fl*^ (*cDdx6*^*–/–*^) and *Ddx6*^*+/+*^ BMDMs without treatment (Media) or treated with LPS plus nigericin (L + N, LPS, 500 ng/mL, 4 h and nigericin, 20 μM, 20 min) for NLRP3 inflammasome activation. **b** Co-IP analysis of endogenous ASC interactions with AIM2, caspase-1, and DDX6 in Lyz2-Cre-expressing *Ddx6*^*fl/fl*^ (*cDdx6*^*–/–*^) and *Ddx6*^*+/+*^ BMDMs without treatment (Media) or transfected with dsDNA (1.5 μg, 1 h) for AIM2 inflammasome activation. **c** Confocal microscopy analysis of DDX6–ASC and ASC–NLRP3 co-localization in Lyz2-Cre-expressing *Ddx6*^*fl/fl*^ (*cDdx6*^*–/–*^) and *Ddx6*^*+/+*^ BMDMs treated with LPS plus nigericin (LPS, 500 ng/mL, 4 h and nigericin, 20 μM, 30 min) for NLRP3 inflammasome activation. The arrows indicate co-localized puncta. Scale bars: 10 μm. **d**, **e** Quantification of ASC speck formation (**d**) and co-localization of DDX6 and ASC, NLRP3 and ASC, and AIM2 and ASC (**e**) in Lyz2-Cre-expressing *Ddx6*^*fl/fl*^ and *Ddx6*^*+/+*^ BMDMs treated with LPS plus nigericin (LPS, 500 ng/mL, 4 h and nigericin, 20 μM, 30 min) or transfected with dsDNA (1.5 μg, 2 h) for NLRP3 and AIM2 inflammasome activation. At least 200 cells were analyzed for each group (*n* = 3 biologically independent samples). **f** BMDMs were pretreated with the DEAD-box RNA helicase inhibitor CR-1-31-B (100 nM) for 2 h and further stimulated for inflammasome activation. Immunoblot analysis of pro-caspase-1 (Pro-Casp1), its subunit p20, and DDX6 in Lyz2-Cre-expressing *Ddx6*^*fl/fl*^ (*cDdx6*^*–/–*^) and *Ddx6*^*+/+*^ BMDMs without treatment (Media) or stimulated with LPS (500 ng/mL, 4 h) and nigericin (20 μM, 60 min), transfected with dsDNA (1.5 μg, 2 h), and infected with *Listeria monocytogenes* (MOI = 50, 3 h), and *Salmonella enterica* Typhimurium (MOI = 3, 2 h) for inflammasome activation. **g** Schematic representation of point mutations in DDX6. **h** Co-IP of endogenous ASC-DDX6 interactions and immunoblot analysis of pro-caspase-1 (Pro-Casp1) and its subunit p20 in Lyz2-Cre-expressing *Ddx6*^*fl/fl*^ (*cDdx6*^*–/–*^) BMDMs transduced with WT DDX6 and point mutations as indicated, without treatment (Media) or further stimulation with LPS plus nigericin (LPS, 500 ng/mL, 4 h and nigericin, 20 μM, 20 min) for NLRP3 inflammasome activation. **i** Co-IP of endogenous ASC-DDX6 interactions and immunoblot analysis of pro-caspase-1 (Pro-Casp1) and its subunit p20 in Lyz2-Cre-expressing *Ddx6*^*fl/fl*^ (*cDdx6*^*–/–*^) BMDMs transduced with WT DDX6 and point mutations as indicated, without treatment (Media) or transfected with dsDNA (1.5 μg, 60 min) for AIM2 inflammasome activation. **j** Proposed model of DDX6-mediated ASC recruitment to NLRP3 and AIM2 discs upon inflammasome activation. Data are from 3 (**e**) or are representative of 3 (**a**–**d**, **f**, **h**) or 2 (**i**) independent experiments with similar results. Data represent the mean ± SEM for (**d**, **e**), 2-sided Student’s *t*-test without multiple-comparisons correction. *P* values are indicated in the graphs.

Inflammasome activation triggers the assembly of insoluble complexes containing ASC, sensor proteins (NLRP3 or AIM2), and pro-caspase-1^27,28^. To determine the step that requires DDX6, we fractionated the lysates of stimulated Lyz2-Cre-expressing *Ddx6*^*fl/fl*^ and *Ddx6*^*+/+*^ BMDMs into soluble and insoluble (pellet) fractions. LPS alone induced the entry of NLRP3 but not ASC or caspase-1 into the pellet, whereas LPS and nigericin recruited NLRP3, ASC, and caspase-1 into the insoluble fraction of WT cells (Supplementary Fig. S3a). DDX6 deficiency markedly reduced the recovery of all three proteins in the pellet (Supplementary Fig. S3a). Similarly, dsDNA transfection (AIM2 activation) drove AIM2, ASC, and caspase-1 into the pellet in WT BMDMs, and this aggregation was impaired in DDX6-deficient cells (Supplementary Fig. S3b). Furthermore, endogenous co-IP assays revealed that ASC and DDX6 interact in WT but not in *Nlrp3*^*–/–*^*Aim2*^*–/–*^ (DKO) BMDMs upon NLRP3 or AIM2 inflammasome activation (Supplementary Fig. S3c, d). Co-immunoprecipitation with anti-ASC antibody confirmed the reduced interactions among ASC, caspase-1, DDX6, and either NLRP3 or AIM2 in DDX6-deficient BMDMs after NLRP3 or AIM2 activation (Fig. 3a, b). Confocal microscopy revealed that DDX6 co-localized with NLRP3 and AIM2 and that ASC speck formation was diminished in DDX6-deficient cells upon NLRP3 or AIM2 inflammasome activation (Fig. 3c–e; Supplementary Fig. S3e, f). These results indicate that DDX6 may regulate the recruitment of ASC to upstream NLRP3 or AIM2 nucleation seeds for ASC speck formation.

The ATPase activity of DDX6 is required for P-body (PB) and stress granule (SG) dynamics, such as PB formation, SG biogenesis, and PB separation from SGs^13–16,29^. To assess its necessity for inflammasome activation, we treated BMDMs with the DEAD-box RNA helicase inhibitor CR-1-31-B^30^. Indeed, the RNA helicase inhibitor CR-1-31-B reduced caspase-1 activation in WT BMDMs triggered by NLRP3 and AIM2 inflammasome activators but not *Salmonella* infection (Fig. 3f). Furthermore, we transduced DDX6-deficient BMDMs with WT DDX6, catalytically inactive point mutants (DDX6^E247Q^ and DDX6^R386E^)^29,31^ and a random control point mutant, DDX6^G34R^, using lentiviruses (Fig. 3g). Compared with that in WT DDX6- and control DDX6^G34R^-transduced BMDMs, caspase-1 activation was reduced in catalytically inactive point mutant DDX6^E247Q^- and DDX6^R386E^-transduced BMDMs upon stimulation with NLRP3 and AIM2 inflammasome activators (Fig. 3h, i). In addition, the interaction between ASC and DDX6 was reduced in DDX6^E247Q^- and DDX6^R386E^-transduced BMDMs but not in WT DDX6- or control DDX6^G34R^-transduced BMDMs in response to NLRP3 and AIM2 inflammasome activation (Fig. 3h, i). Consistent with these findings, the interactions between ASC and caspase-1 and the upstream sensors NLRP3 and AIM2 were also inhibited in DDX6^E247Q^- and DDX6^R386E^-transduced BMDMs compared with WT DDX6- or control DDX6^G34R^-transduced BMDMs (Supplementary Fig. S3g, h). Collectively, these data establish that DDX6 uses its RNA helicase activity to recruit ASC to the NLRP3 and AIM2 platforms, enabling ASC-speck assembly and inflammasome activation (Fig. 3j).

### ASC specks assemble in P-bodies and transit to stress granules during inflammasome activation

**Fig. 4:**
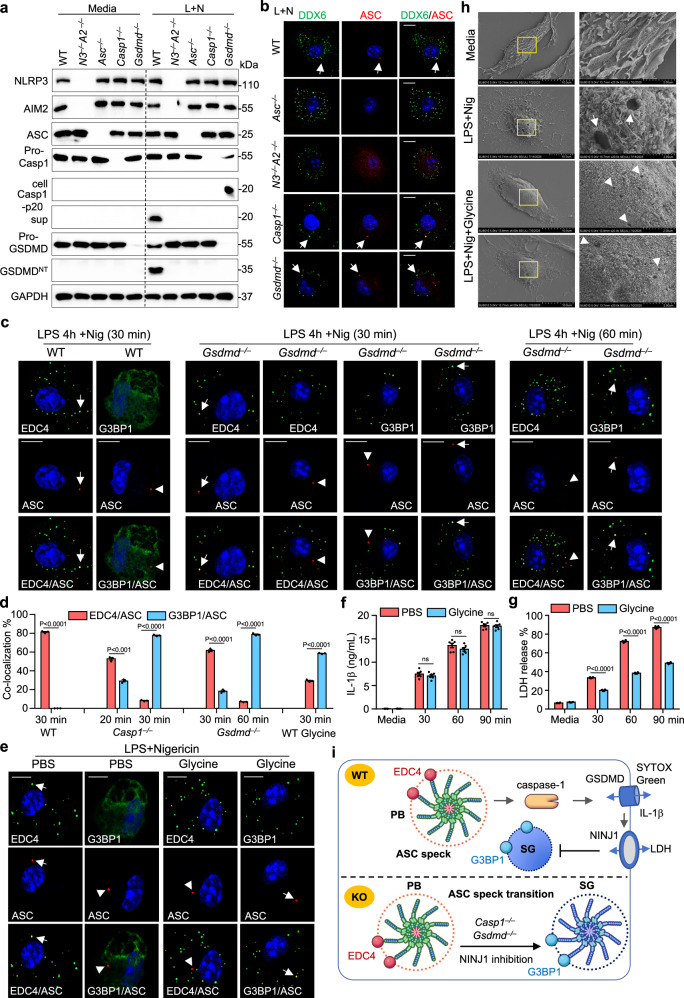
ASC specks co-localize with PBa in WT BMDMs and with SGs in pyroptosis-blocked BMDMs. **a** Immunoblot analysis of pro-caspase-1 (Pro-Casp1), its subunit p20, full-length and cleaved GSDMD (GSDMD^NT^), NLRP3, AIM2, and ASC in WT, *Nlrp3*^*–/–*^*Aim2*^*–/–*^ (*N3*^*–/–*^*A2*^*–/–*^), *Asc*^*–/–*^, *Caspase-1*^*–/–*^ (*Casp1*^*–/–*^), and *Gsdmd*^*–/–*^ BMDMs without treatment (Media) or stimulated with LPS plus nigericin (LPS, 500 ng/mL, 4 h and nigericin, 20 μM, 30 min) for NLRP3 inflammasome activation. **b** Confocal microscopy analysis of DDX6 and ASC co-localization in WT, *Nlrp3*^*–/–*^*Aim2*^*–/–*^ (*N3*^*–/–*^*A2*^*–/–*^), *Asc*^*–/–*^, *Caspase-1*^*–/–*^ (*Casp1*^*–/–*^), and *Gsdmd*^*–/–*^ BMDMs stimulated with LPS plus nigericin (LPS, 500 ng/mL, 4 h and nigericin, 20 μM, 30 min) for NLRP3 inflammasome activation. The arrows indicate co-localized puncta. Scale bars: 10 μm. **c** Confocal microscopy analysis of ASC co-localization with EDC4 or G3BP1 in WT and *Gsdmd*^*–/–*^ BMDMs stimulated with LPS plus nigericin (LPS, 500 ng/mL, 4 h and nigericin, 20 μM, 30 min and 60 min) for NLRP3 inflammasome activation. The arrows indicate co-localized puncta, and the arrowheads indicate puncta that are not co-localized. Scale bars: 10 μm. **d** Quantification of the co-localization of EDC4 and ASC, G3BP1 and ASC in WT, *Caspase-1*^*–/–*^ (*Casp1*^*–/–*^), and *Gsdmd*^*–/–*^ BMDMs and WT BMDMs treated with glycine (5 mM) and further stimulated with LPS plus nigericin (LPS, 500 ng/mL, 4 h and nigericin, 20 μM, 20 min, 30 min, and 60 min) for NLRP3 inflammasome activation. In the glycine-treated group, the co-localization of ASC specks with EDC4 or G3BP1 puncta was analyzed in at least 300 ASC speck-positive cells per group. For the other groups, at least 150 ASC speck-positive cells per group were analyzed for co-localization with EDC4 or G3BP1 at each time point (*n* = 3 biologically independent samples). **e** Confocal microscopy analysis of ASC co-localization with EDC4 or G3BP1 in WT BMDMs treated with glycine (5 mM) and further stimulated with LPS plus nigericin (LPS, 500 ng/mL, 4 h and nigericin, 20 μM, 30 min) for NLRP3 inflammasome activation. The arrows indicate co-localized puncta, and the arrowheads indicate puncta that are not co-localized. Scale bars: 10 μm. **f**, **g** Analysis of IL-1β release (**f**) and LDH secretion (**g**) in WT BMDMs treated with glycine (5 mM) and further stimulated with LPS plus nigericin (LPS, 500 ng/mL, 4 h and nigericin, 20 μM, 30 min, 60 min, and 90 min) for NLRP3 inflammasome activation. **h** Scanning electron microscopy (SEM) analysis of WT BMDMs treated with glycine (5 mM) and further stimulated with LPS plus nigericin (LPS, 500 ng/mL, 4 h and nigericin, 20 μM, 45 min) for NLRP3 inflammasome activation. The arrows indicate the large membrane pores, and the arrowheads indicate the small membrane pores. Scale bars: 10 μm for the left and 2 μm for the right. **i** Proposed model of ASC speck formation in PBs and the transition to SGs in pyroptosis-blocked BMDMs (KO) during inflammasome activation. Data are from 3 (**d**, **f**, **g**) or are representative of 3 (**a**–**c**, **e**, **h**) independent experiments with similar results. Data represent the mean ± SEM for (**d**, **f**, **g**), 2-sided Student’s *t*-test without multiple-comparisons correction. *P* values are indicated in the graphs.

DDX6 is required for normal PB assembly and the biogenesis of SGs^13–15^, and PBs have been linked to inflammasome control, whereas SGs can inhibit death pathways^17,32,33^. Therefore, we examined the spatial relationships of ASC specks with PBs and SGs. After NLRP3 inflammasome stimulation (LPS and nigericin), ASC specks formed in WT, *Caspase-1*^*–/–*^, and *Gsdmd*^*–/–*^ BMDMs but were absent from *Nlrp3*^*–/–*^*Aim2*^*–/–*^ and *Asc*^*–/–*^ cells (Fig. 4a, b), confirming that speck formation depends on the sensor and ASC itself, not on downstream caspase-1 or GSDMD. We further investigated the co-localization of ASC speck and DDX6 in WT, *Caspase-1*^*–/–*^, and *Gsdmd*^*–/–*^ BMDMs. Notably, the deficiency of caspase-1 or GSDMD in BMDMs did not reduce the co-localization of DDX6 and ASC speck (Fig. 4b).

We then comprehensively characterized the intracellular distribution of EDC4 (PB marker) and G3BP1 (SG marker)^13,14^ in WT, *Nlrp3*^*–/–*^*Aim2*^*–/–*^, *Asc*^*–/–*^, *Caspase-1*^*–/–*^, and *Gsdmd*^*–/–*^ BMDMs in response to cellular stress for SG formation (LPS plus sodium arsenite, LPS + SA)^17^ and inflammasome activation. The puncta distribution of EDC4 (PBs formation) was observed in both WT and knockout BMDMs, including *Nlrp3*^*–/–*^*Aim2*^*–/–*^, *Asc*^*–/–*^, *Caspase-1*^*–/–*^, and *Gsdmd*^*–/–*^, with and without treatment (Supplementary Fig. S4a–c). Cellular stress SA-induced SGs assembled equally (large G3BP1 puncta) in WT and knockout BMDMs (Supplementary Fig. S4a). In contrast to the canonical SGs induced by SA, the inflammasome triggered smaller G3BP1-positive SGs in the absence of a functional inflammasome (in *Nlrp3*^*–/–*^*Aim2*^*–/–*^, *Asc*^*–/–*^, *Caspase-1*^*–/–*^, and *Gsdmd*^*–/–*^ BMDMs) (Supplementary Fig. S4b, c). These findings indicate that the inhibition of inflammasome signaling is associated with SG formation and that the downstream event mediated by GSDMD may be a determining factor for SG assembly. Notably, under all the conditions tested, EDC4 and G3BP1 formed distinct puncta that were largely segregated (Supplementary Fig. S4a–c).

We then analyzed the co-localization of ASC specks with PBs and SGs in WT, *Caspase-1*^*–/–*^, and *Gsdmd*^*–/–*^ BMDMs during LPS plus nigericin treatment. In WT BMDMs, 81.4% of ASC specks co-localized with EDC4-positive PBs 20–30 min after nigericin treatment (Fig. 4c, d). In *Caspase-1*^*–/–*^ cells, the fraction of ASC specks co-localized with EDC4-positive PBs decreased from 52.8% to 8.3% between 20 and 30 min, whereas that co-localized with G3BP1-positive SGs increased from 29.3% to 77.7% (Fig. 4d; Supplementary Fig. S5a). A similar temporal shift was observed in *Gsdmd*^*–/–*^ BMDMs: ASC specks relocated from EDC4-positive PBs (61.7% at 30 min) to G3BP1-positive SGs (78.7% at 60 min) (Fig. 4c, d). In addition, the G3BP1-positive puncta in the *Gsdmd*^*–/–*^ BMDMs were co-localized with another stress granule marker, ATXN2L^34^, following treatment with LPS plus nigericin for 30 and 60 min (Supplementary Fig. S5b). NEK7, an essential NLRP3-interacting protein for inflammasome assembly^6^, was also investigated. Endogenous co-IP analysis of anti-ASC in *Gsdmd*^*–/–*^ BMDMs revealed that a complex containing ASC, DDX6, NLRP3, and NEK7 preferentially interacts with EDC4 and G3BP1 at 30 min and 60 min post-treatment (Supplementary Fig. S5c). Confocal microscopy further confirmed the co-localization of DDX6 with ASC, EDC4 with NEK7, and G3BP1 with NEK7 (Supplementary Fig. S5d). Collectively, these data indicate that in the absence of caspase-1 or GSDMD, ASC specks together with DDX6 and inflammasome receptors originate in PBs and subsequently relocate to SGs during inflammasome activation.

To test whether membrane integrity governs SG formation, we treated WT BMDMs with glycine, which blocks NINJ1-dependent plasma membrane rupture during pyroptosis, post-apoptosis lysis, and necroptosis^35–38^. Glycine induced the formation of G3BP1-positive SGs in WT cells, with 58.6% of ASC specks co-localizing with SGs and 29.4% of ASC specks with PBs at 30 min after LPS plus nigericin treatment (Fig. 4d, e). Glycine treatment did not affect IL-1β release (Fig. 4f), the cleavage of caspase-1 and GSDMD (Supplementary Fig. S5e) or death kinetics (Supplementary Fig. S5f, g). However, it inhibited the LDH release (Fig. 4g) triggered by LPS plus nigericin. Electron microscopy revealed large membrane lesions in control but not glycine-treated BMDMs in response to LPS plus nigericin (Fig. 4h), which is consistent with previous reports that NINJ1 mediates plasma membrane rupture and is crucial for the secretion of high-molecular-weight proteins^36,37^. Therefore, the preservation of membrane integrity is associated with SG formation and facilitates the transition of ASC specks from PBs to SGs during inflammasome activation (Fig. 4i).

### DDX6 governs ASC speck formation in PBs and its relocation to SGs during inflammasome activation

**Fig. 5:**
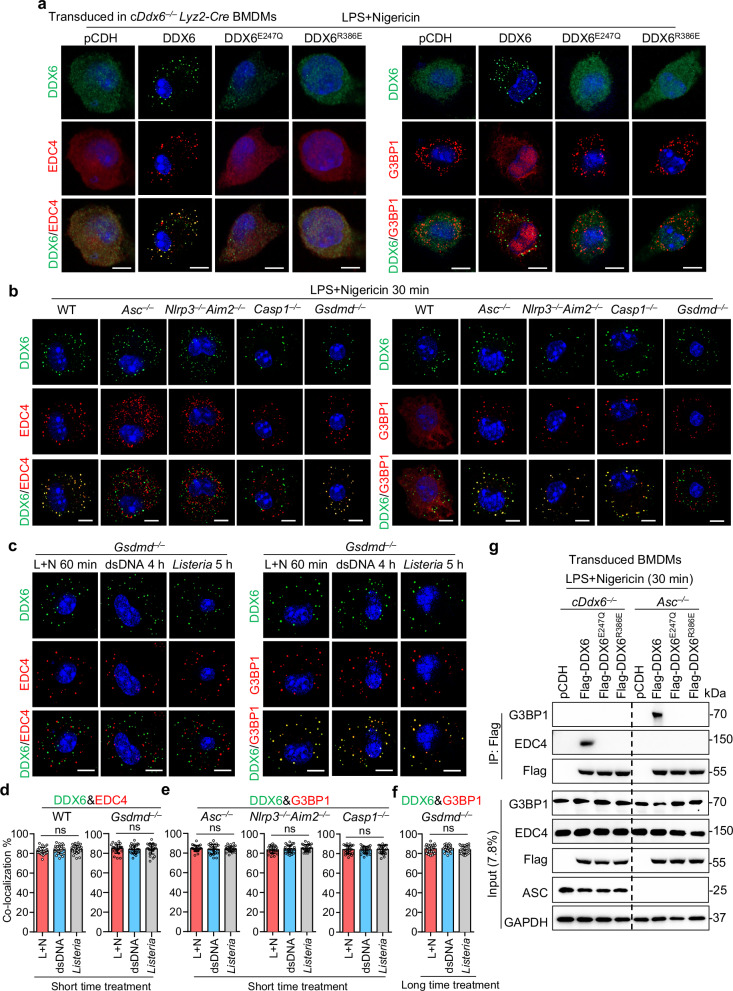
DDX6 mediates ASC speck formation in PBs and the transition to SGs. **a** Confocal microscopy analysis of DDX6 co-localization with EDC4 or G3BP1 in Lyz2-Cre-expressing *Ddx6*^*fl/fl*^ (*cDdx6*^*–/–*^) BMDMs transduced with WT DDX6 or the indicated point mutants, followed by treatment with LPS plus nigericin (LPS, 500 ng/mL, 4 h and nigericin, 20 μM, 30 min) for NLRP3 inflammasome activation. Scale bars: 10 μm. **b** Confocal microscopy analysis of DDX6 co-localization with EDC4 or G3BP1 in WT, *Nlrp3*^*–/–*^*Aim2*^*–/–*^, *Asc*^*–/–*^, *Caspase-1*^*–/–*^ (*Casp1*^*–/–*^), and *Gsdmd*^*–/–*^ BMDMs stimulated with LPS plus nigericin (LPS, 500 ng/mL, 4 h and nigericin, 20 μM, 30 min) for NLRP3 inflammasome activation. Scale bars: 10 μm. **c** Confocal microscopy analysis of DDX6 co-localization with EDC4 or G3BP1 in *Gsdmd*^*–/–*^ BMDMs stimulated with LPS plus nigericin (LPS, 500 ng/mL, 4 h and nigericin, 20 μM, 60 min), transfected with dsDNA (1.5 μg, 4 h), or infected with *Listeria monocytogenes* (MOI = 50, 5 h) for inflammasome activation. Scale bars: 10 μm. **d** Quantification of DDX6-EDC4 co-localization in WT and *Gsdmd*^*–/–*^ BMDMs stimulated with LPS plus nigericin (LPS, 500 ng/mL, 4 h and nigericin, 20 μM, 30 min), transfected with dsDNA (1.5 μg, 2 h), or infected with *Listeria monocytogenes* (MOI = 50, 2 h). *n* = 25 for each group. **e** Quantification of DDX6-G3BP1 co-localization in *Asc*^*–/–*^, *Nlrp3*^*–/–*^*Aim2*^*–/–*^, and *Caspase-1*^*–/–*^ (*Casp1*^*–/–*^) BMDMs under the same conditions as in **d**. *n* = 25 for each group. **f** Quantification of DDX6-G3BP1 co-localization in *Gsdmd*^*–/–*^ BMDMs stimulated with LPS plus nigericin (LPS, 500 ng/mL, 4 h and nigericin, 20 μM, 60 min), transfected with dsDNA (1.5 μg, 4 h), or infected with *Listeria monocytogenes* (MOI = 50, 5 h). *n* = 25 for each group. **g** Co-IP analysis of DDX6 interactions with EDC4 and G3BP1 in Lyz2-Cre-expressing *Ddx6*^*fl/fl*^ and *Asc*^*–/–*^ BMDMs transduced with WT DDX6 or the indicated point mutants, followed by stimulation with LPS plus nigericin (LPS, 500 ng/mL, 4 h and nigericin, 20 μM, 30 min) for NLRP3 inflammasome activation. Data are representative of 3 (**a**–**f**) or 2 (**g**) independent experiments with similar results. Data represent the mean ± SEM for **d**–**f**, 2-sided Student’s *t*-test without multiple comparisons correction.

To determine whether DDX6 controls ASC speck formation in PBs upon inflammasome activation, we stimulated Lyz2-Cre-expressing *Ddx6*^*fl/fl*^ and *Ddx6*^*+/+*^ BMDMs with LPS plus nigericin, dsDNA transfection, or *Listeria* infection (Supplementary Fig. S6a). EDC4-positive PBs were almost undetectable in DDX6-deficient cells under basal or stimulated conditions, whereas G3BP1-positive SGs formed spontaneously in knockout cells upon inflammasome induction (Supplementary Fig. S6a). We transduced DDX6-deficient BMDMs with WT DDX6 or the catalytically inactive point mutants DDX6^E247Q^ and DDX6^R386E^ and performed co-localization analysis of DDX6 and EDC4 or G3BP1 in response to LPS plus nigericin. Consistent with previous studies showing that DDX6 RNA helicase activity is required for P-body assembly^15^, WT DDX6, but not the catalytically inactive mutants DDX6^E247Q^ or DDX6^R386E^, restored the number of EDC4 puncta and suppressed the formation of aberrant G3BP1 puncta in DDX6-deficient BMDMs (Fig. 5a; Supplementary Fig. S6b). WT DDX6 co-localized with EDC4 puncta, but the mutants DDX6^E247Q^ and DDX6^R386E^ failed to accumulate in SGs (Fig. 5a), indicating that helicase activity is required for DDX6 puncta formation, PB assembly, and recruitment to SGs.

We next examined the distribution of DDX6 in pyroptosis-competent and pyroptosis-deficient cells. After brief exposure to NLRP3 or AIM2 stimuli (30 min nigericin, 2 h dsDNA, 3 h *Listeria*), DDX6 co-localized primarily with EDC4 in WT and *Gsdmd*^*–/–*^ BMDMs but with G3BP1 in *Nlrp3*^*–/–*^*Aim2*^*–/–*^, *Asc*^*–/–*^, and *Caspase-1*^*–/–*^ cells (Fig. 5b, d, e; Supplementary Fig. S6c, d). Prolonged stimulation (60 min of nigericin, 4 h of dsDNA, and 5 h of *Listeria* infection) prompted DDX6 to shift from EDC4 to G3BP1 puncta in *Gsdmd*^*–/–*^ BMDMs (Fig. 5c, f). Co-IP analysis of transduced BMDMs further confirmed that WT DDX6, but not DDX6^E247Q^ or DDX6^R386E^ interacted with EDC4 in WT BMDMs and with G3BP1 in *Asc*^*–/–*^ BMDMs during inflammasome activation (Fig. 5g). Collectively, these results show that DDX6 enables ASC speck formation by sustaining PB assembly and, when pyroptosis is blocked, drives the subsequent relocation of ASC specks to SGs during inflammasome activation.

### DDX6 undergoes LLPS to recruit ASCs and activate inflammasomes

DEAD-box (DDX) RNA helicases are global regulators of phase-separated membraneless organelles, such as PBs and SGs^23^. Using IUPRED2 (https://iupred2a.elte.hu/), we predicted that DDX6 contains an intrinsically disordered region (IDR) at its N-terminus (residues 1–90), suggesting its potential for phase separation^39^ (Supplementary Fig. S7a, b). To confirm the role of the IDR of DDX6 in phase separation, we performed an optoDroplet assay by fusing the IDR, Domain 1, and Domain 2 of DDX6 with mCherry and Cry2 — a light-sensitive *Arabidopsis thaliana* protein that promotes LLPS upon blue light exposure (Supplementary Fig. S7b, c)^40^. Notably, only the mCherry-Cry2-IDR fusion (not Domain 1, Domain 2, or mCherry-Cry2 alone) exhibited rapid blue light-dependent clustering and puncta formation (Supplementary Fig. S7c), indicating that the IDR of DDX6 is essential for LLPS.

**Fig. 6:**
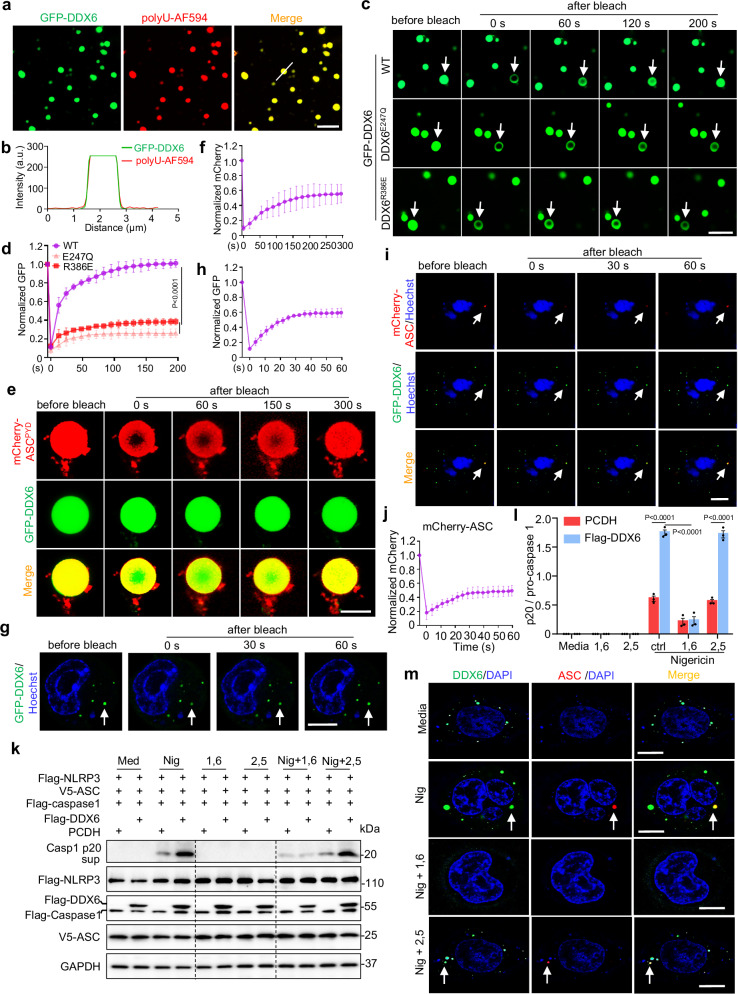
DDX6 undergoes LLPS to promote ASC recruitment. **a** Representative micrographs of GFP-DDX6 droplets (20 μM) and Alexa Fluor 594-labeled polyU RNA droplets (60 ng/μL) in the presence of ATP (10 mM). Scale bar: 5 μm. **b** Quantitative line profile of GFP-DDX6 and polyU-AF594 co-localization along the white line in **a**. **c**, **d** Representative micrographs (**c**) and quantification (**d**) of GFP fused with WT DDX6 or mutant (DDX6^E247Q^ and DDX6^R386E^) droplets (20 μM) undergoing fluorescence recovery after photobleaching (FRAP) for 200 s in the presence of RNA (60 ng/μL) and ATP (10 mM). The arrows indicate bleached droplets. Scale bar: 5 μm for **c**. **e**, **f** Representative micrographs (**e**) and quantification (**f**) of mCherry-ASC^PYD^ droplets (20 μM) co-incubated with GFP-DDX6 (20 μM), RNA (60 ng/μL), and ATP (10 mM) during FRAP for 300 s. Scale bar: 1 μm for **e**. **g**, **h** Representative micrographs (**g**) and quantification (**h**) of GFP-DDX6 puncta undergoing FRAP over 60 s in HEK293T cells transfected with GFP-DDX6. The arrows indicate bleached puncta. Scale bar: 10 μm for **g**. **i**, **j** Representative micrographs (**i**) and quantification (**j**) of mCherry-ASC puncta after undergoing FRAP for 60 s in HEK293T cells transfected with GFP-DDX6, mCherry-ASC, NLRP3, and Caspase-1, followed by nigericin treatment (20 μM, 30 min). **k** Immunoblot analysis of pro-caspase-1 (Pro-Casp1), its subunit p20, NLRP3, DDX6, and ASC in HEK293T cells transfected with FLAG-DDX6, V5-ASC, Flag-NLRP3, and Flag-Caspase-1 and treated with nigericin (20 μM, 30 min) in the presence or absence of 1,6-hexanediol (1,6; 5%) or 2,5-hexanediol (2,5; 5%). **l** Quantification of caspase-1 activation (p20 cleavage) from **k**. **m** Confocal microscopy of DDX6 and ASC expression in HEK293T cells transfected with FLAG-DDX6, V5-ASC, Flag-NLRP3, and Flag-Caspase-1 and treated with nigericin (20 μM, 30 min) in the presence or absence of 1,6-hexanediol (1,6; 5%) or 2,5-hexanediol (2,5; 5%). Data are from 3 (**d**, **f**, **h**, **j**, **l**) or are representative of 3 independent experiments with similar results (**a**, **b**, **c**, **e**, **g**, **i**, **k**, **m**). Data represent the mean ± SEM for (**d**, **l**), 2-sided Student’s *t*-test without multiple-comparisons correction. *P* values are indicated in the graphs.

To determine whether full-length DDX6 undergoes phase separation, we purified recombinant GFP-DDX6. In the presence of RNA and ATP, GFP-DDX6 formed spherical droplets at high protein concentrations (up to 20 μM), whereas a high salt concentration (300 mM) significantly suppressed droplet formation (Supplementary Fig. S7d, e). Using polyU RNA labeled with Alexa Fluor 594 (polyU-AF594), we confirmed that RNA is required for GFP-DDX6 droplet formation, as GFP-DDX6 and polyU-AF594 co-localized completely (Fig. 6a, b). Fluorescence recovery after photobleaching (FRAP) analysis revealed rapid recovery in GFP fused with WT DDX6 droplets but not in those of the helicase-deficient mutants DDX6^E247Q^ or DDX6^R386E^ (Fig. 6c, d), suggesting that RNA helicase activity is critical for efficient LLPS.

Next, we investigated whether DDX6 recruits ASC via phase separation. We purified mCherry-ASC^PYD^ and mCherry-ASC^CARD^ fusion proteins and found that, unlike GFP-DDX6, they spontaneously formed irregular aggregates with filamentous structures in solution (Supplementary Fig. S8a). mCherry-ASC^PYD^ also exhibited large filamentous structures (Supplementary Fig. S8a). FRAP experiments revealed no fluorescence recovery in mCherry-ASC^PYD^ or mCherry-ASC^CARD^ aggregates (Supplementary Fig. S8b). Time-lapse imaging revealed that mCherry-ASC^PYD^ but not mCherry-ASC^CARD^ co-localized with most of the GFP-DDX6 droplets (Supplementary Fig. S8c, d). Moreover, the DDX6 mutants DDX6^E247Q^ and DDX6^R386E^ failed to recruit mCherry-ASC^PYD^ (Supplementary Fig. S8d). After photobleaching, the mCherry-ASC^PYD^ within the GFP-DDX6 condensates rapidly recovered (Fig. 6e, f).

To assess whether DDX6 drives LLPS and ASC speck formation in cells, we transfected HEK293T cells with GFP-DDX6 plasmid with and without the mCherry-ASC plasmid, followed by nigericin stimulation and FRAP analysis. Live-cell imaging revealed that GFP-DDX6 condensates recovered 60% of their initial fluorescence within 60 s post bleaching (Fig. 6g, h), confirming their liquid-like properties. In cells co-expressing GFP-DDX6, mCherry-ASC, NLRP3, and caspase-1, nigericin induced ASC speck formation, which co-localized with GFP-DDX6 puncta (Fig. 6i). Additionally, GFP-DDX6 accelerated mCherry-ASC condensation after photobleaching (Fig. 6i, j).

To further validate the role of DDX6 in inflammasome assembly, we reconstituted the NLRP3 inflammasome in HEK293T cells by co-expressing V5-ASC, Flag-NLRP3, and Flag-caspase-1 with and without Flag-DDX6 (Fig. 6k). Upon nigericin treatment, the activation of caspase-1 by DDX6 was significantly inhibited by the LLPS disruptor 1,6-hexanediol (1,6-HD) but not its inactive analog 2,5-hexanediol (2,5-HD)^41^ (Fig. 6k, l). Consistently, 1,6-HD (but not 2,5-HD) dissolved DDX6 puncta and ASC specks (Fig. 6m). Together, these findings demonstrate that DDX6 promotes ASC compartmentalization via LLPS to facilitate inflammasome assembly.

### DDX6 promotes host defense against *Listeria* infection

**Fig. 7:**
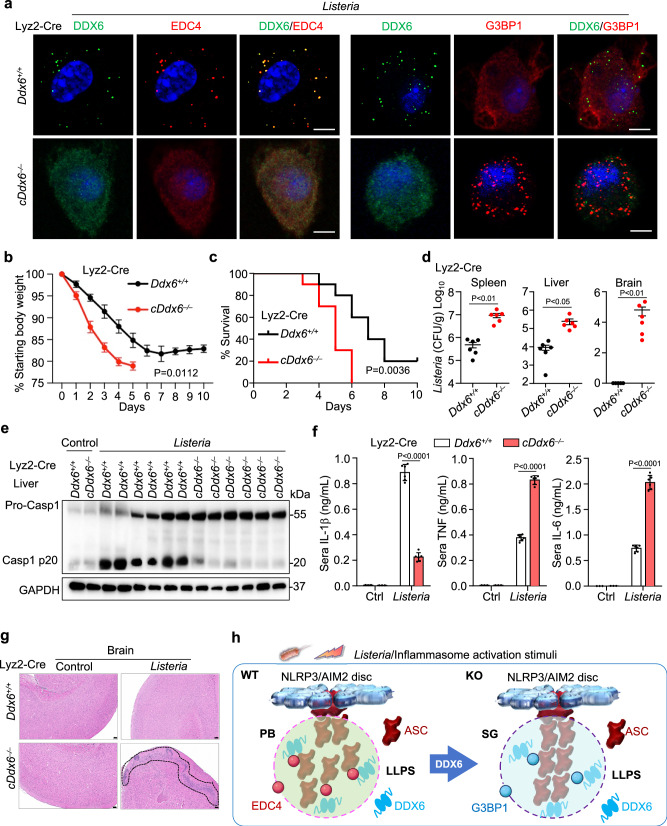
DDX6 promotes host defense against *Listeria* infection. **a** Confocal microscopy analysis of DDX6 co-localization with EDC4 or G3BP1 in Lyz2-Cre-expressing *Ddx6*^*fl/fl*^ (*cDdx6*^*–/–*^) and *Ddx6*^*+/+*^ BMDMs infected with *Listeria monocytogenes* (MOI = 50, 2 h). Scale bars: 10 μm. **b**, **c** Lyz2-Cre-expressing *Ddx6*^*fl/fl*^ (*cDdx6*^*–/–*^, *n* = 10) and littermate control *Ddx6*^*+/+*^ (*n* = 10) female mice were intraperitoneally infected with *Listeria monocytogenes* (6.0 × 10^5^ CFUs per mouse), and body weights (**b**) and survival (**c**) were monitored. **d** Bacterial burdens in the spleen, liver, and brain on Day 2 post infection in Lyz2-Cre-expressing *Ddx6*^*fl/fl*^ (*n* = 6) and littermate control *Ddx6*^*+/+*^ (*n* = 6) female mice infected as in **b**. **e** Immunoblot analysis of pro-caspase-1 (Pro-Casp1) and its subunit p20 in livers from uninfected and *Listeria monocytogenes*-infected mice in **d**. **f** ELISA of IL-1β, TNF, and IL-6 in sera from uninfected and *Listeria monocytogenes*-infected mice in **d**. **g** H&E staining of brain sections from uninfected and *Listeria monocytogenes*-infected mice in **d**. Dashed outlines indicate immune cell infiltrates. Scale bars: 100 μm. **h** Proposed model: DDX6 mediates ASC speck formation in PBs via LLPS, facilitates their transition to SGs, and promotes inflammasome activation to enhance host defense against *L. monocytogenes*. The data are representative of 3 independent experiments with similar results (**a**–**g**). Data represent the mean ± SEM for (**d**, **f**), 2-sided Student’s *t*-test without multiple comparisons correction, two-way ANOVA for **b**, and log-rank (Mantel–Cox) test for **c**. *P* values are indicated in the graphs.

Inflammasome activation is critical for host defense against bacterial infections^42^. *Listeria monocytogenes* can cause life-threatening conditions such as meningitis and sepsis, particularly in immunocompromised individuals^43^. Because *Listeria* infection triggers both NLRP3 and Aim2 inflammasome activity^44^, we examined the role of DDX6 in Lyz2-Cre-expressing *Ddx6*^*fl/fl*^ and *Ddx6*^*+/+*^ BMDMs and mice. Confocal microscopy revealed that DDX6 deficiency disrupted PB assembly and induced SG formation upon *Listeria* infection, LPS treatment, nigericin treatment, or dsDNA transfection (Fig. 7a; Supplementary Fig. S9a). In contrast, DDX6 co-localized with EDC4 (a PB marker) in WT BMDMs during inflammasome activation (Fig. 7a; Supplementary Fig. S9a), suggesting that DDX6 facilitates ASC speck formation in response to *Listeria* infection.

To assess the role of DDX6 in bacterial defense, we intraperitoneally infected Lyz2-Cre-expressing *Ddx6*^*fl/fl*^ mice and littermate *Ddx6*^*+/+*^ mice with a lethal dose of *Listeria monocytogenes* (6.0 × 10⁵ CFU). By Day 5, *Ddx6*^*fl/fl*^ mice exhibited greater weight loss (78.9% vs. 84.8% of the initial weight) and succumbed entirely by Day 6, whereas 20% of *Ddx6*^*+/+*^ mice survived beyond Day 10 (Fig. 7b, c). To determine whether increased mortality correlated with bacterial burden, we quantified *Listeria* loads in organs two days post-infection. Compared with *Ddx6*^+/+^ mice, *Ddx6*^*fl/fl*^ mice had significantly higher bacterial counts in the spleen, liver, and brain (Fig. 7d). Notably, *Ddx6*^*+/+*^ mice showed minimal brain infection (Fig. 7d), underscoring the importance of DDX6 in preventing neuroinvasion. Consistent with impaired inflammasome activation, *Ddx6*^*fl/fl*^ livers exhibited reduced caspase-1 cleavage (Fig. 7e) and lower serum IL-1β levels (Fig. 7f). Conversely, *Ddx6*^*fl/fl*^ mice produced higher levels of IL-6 and TNF, likely due to uncontrolled bacterial proliferation (Fig. 7f). Histopathology revealed exacerbated immune cell infiltration in *Ddx6*^*fl/fl*^ livers, spleens, and brains (Fig. 7g). In summary, DDX6 promotes ASC speck assembly and inflammasome activation by recruiting ASC via phase separation, a mechanism that is integral to its role in host defense against *Listeria* infection (Fig. 7h).

## Discussion

ASC recruitment and speck formation are critical for NLRP3 and AIM2 inflammasome activation. Cryo-EM structures demonstrate that NLRP3 discs and AIM2 filaments serve as nucleation seeds for ASC recruitment and elongation^4–6^, and the mechanisms governing cytoplasmic ASC speck condensation remain under active investigation^45,46^. Consistent with previous findings that DDX6 functions as a scaffold protein for P-body assembly^34^, our study demonstrated that it promotes ASC speck formation within P-bodies via LLPS. DDX6 compartmentalizes ASC specks to facilitate caspase-1 activation and downstream signaling. Intriguingly, in cells lacking caspase-1, GSDMD, or NINJ1, DDX6 mediates ASC speck translocation to stress granules upon inflammasome activation. These findings establish endogenous ASC specks as dynamic membraneless organelles that localize to P-bodies or SGs in a mutually exclusive manner, governed by the protein interaction hub DDX6.

LLPS enables cells to organize membraneless organelles with diverse functions through the formation of biomolecular condensates. Recent work by Zou et al. identified NLRP3 palmitoylation by ZDHHC7 and its IDR as key drivers of NLRP3 condensation via phase separation^47,48^, providing a unifying mechanism for upstream inflammasome activation signals. ASC speck formation involves oligomerization of the ASC^PYD^ domain into filaments, followed by ASC^CARD^-mediated condensation, which results in the recruitment and activation of caspase-1 through CARD-CARD interactions^49,50^. Our data place DDX6 downstream of NLRP3/AIM2 oligomerization, where it facilitates ASC filament elongation via phase separation to drive inflammasome assembly. The reversible nature of phase-separated ASC specks may enable rapid caspase-1 recruitment and efficient stress responses.

ASC specks have been described as perinuclear puncta associated with various membranous organelles, such as the endoplasmic reticulum, trans-Golgi network, and endosomal vesicles^22,46,51^. Here, we show that ASC specks co-localize with membraneless organelle P-bodies (in WT cells) or SGs (in caspase-1/GSDMD/NINJ1-deficient cells). P-bodies and SGs are evolutionarily conserved ribonucleoprotein (RNP) granules formed via translational repression^9,52^. P-bodies increase mRNA decay machinery, whereas SGs sequester stalled translation preinitiation complexes^11,53–55^. Both exhibit dynamic, LLPS-driven architectures, with dense cores surrounded by liquid-phase shells^54,56,57^. The co-localization of ASC specks with RNP granules aligns with recent nanoscale analyses revealing ASC specks as biomolecular condensates with dense cores and filamentous peripheries^45^. This liquid-like state likely enables rapid, reversible assembly under stress.

Dynamic P-bodies and SGs can form transient docking interactions and exchange components^13,14^. DEAD-box ATPases remodel RNA‒protein interactions within membraneless organelles, acting as scaffolds (in cis) or disruptors (in trans)^23,58–60^. Their ATPase activity and RNA binding are critical for regulating P-body and SG dynamics^24,56,61^. DDX6 (yeast Dhh1) orchestrates P-body assembly by recruiting components and limiting SG interactions during stress^13,14,34^. We demonstrated that DDX6 interacts with EDC4 (in P-bodies) and G3BP1 (in SGs) in WT and caspase-1-GSDMD-NINJ1-deficient BMDMs, respectively. Like its mutually exclusive partnerships with EDC3 and Tral^62^, DDX6 acts as an interaction hub to direct ASC speck localization to P-bodies or SGs, depending on the recruitment of specific interactions^63^. Although p62-dependent P-bodies (pd-PBs) have recently been linked to NLRP3 activation via p62–DDX6 interactions^33^, the signals that drive the specificity of DDX6 puncta co-localization with ASC specks and facilitate their transition between RNP granules remain unknown.

The relationship between inflammasomes and SGs is complex. Early studies suggested that SGs negatively regulate inflammasomes by competing for DDX3X, which binds NLRP3 to balance cell death/survival^17^. Conversely, shared components and potassium efflux (an NLRP3 trigger) may inhibit SG assembly^32^. Our data reveal that ASC specks and SGs coexist in caspase-1–GSDMD–NINJ1-deficient cells and that membrane integrity may be critical for SG formation. Upon inflammasome stimulation, complexes such as NLRP3–ASC or AIM2–ASC can form non-canonical platforms that recruit and activate caspase-8. These platforms serve as a backup cell death system, shifting cell death from pyroptosis to apoptosis in cells deficient in caspase-1 or GSDMD^64–66^. Furthermore, PANoptosis is a highly coordinated and flexible cell death process with a built-in fail-safe mechanism that is mediated by PANoptosomes. These complexes rely on the scaffold protein ASC to engage with distinct components and simultaneously activate multiple cell death pathways^67^. Notably, intracellular ASC specks can also be secreted into the extracellular space and subsequently internalized by bystander cells following inflammasome activation, thereby amplifying the inflammatory response^68,69^. Here, we report that ASC specks are translocated to SGs in the absence of caspase-1 or GSDMD. ASC specks within SGs may facilitate alternative caspase activation or stabilization, a possibility that warrants further investigation. Our work identifies DDX6 as a central regulator of inflammasome activation, highlighting its role beyond LLPS in dynamically localizing ASC specks to P-bodies (for formation) or SGs (for maintenance). These findings offer new therapeutic avenues for inflammasome-related diseases.

## Materials and methods

### Mice

*Ddx6*^*fl/fl*^ mice were generated by Cyagen Biosciences, Inc. Exons 3 and 4 of the *Ddx6* gene were knocked out using the CRISPR-Cas9 system (Supplementary Fig. S2a). *Aim2*^*–/–*^ mice were provided by F. Shao (National Institute of Biological Sciences, Beijing, China); *Asc*^*–/–*^ and *Nlrp3*^*–/–*^ mice were provided by D. Wang (Zhejiang University, Hangzhou, China); *Caspase-1*^*–/–*^ mice were provided by S. Zhu (University of Science and Technology of China, Hefei, China); and *Gsdmd*^*–/–*^ mice were provided by L. Sun (Fudan University, Shanghai, China). *Nlrp3*^*–/–*^*Aim2*^*–/–*^ mice were generated by crossing *Nlrp3*^*–/–*^ and *Aim2*^*–/–*^ mice as previously described^70^. *Ddx6*^*fl/fl*^ mice were crossed with Lyz2-Cre mice to generate macrophage-specific conditional knockout mice as previously described^42^. WT and knockout mice were SPF-clean and maintained under specific pathogen-free conditions at the Animal Resource Center at Shandong University, Jinan, Shandong Province, China. Our research complies with all relevant ethical regulations. The animal experiments were conducted under the approval of the Ethics Committee of Scientific Research of Shandong University (IACUC) with approval number ECSBMSSDU2021-2-171.

### Preparation of BMDMs, treatment, and bacterial infection

To generate BMDMs, bone marrow (BM) cells were cultured for 5 days in L929-conditioned DMEM/F-12 supplemented with 10% FBS, 1% non-essential amino acids, and 1% penicillin‒streptomycin. An RNA helicase inhibitor (MCE, HY-136453) was obtained from MedChemExpress. WT and knockout BMDMs were pretreated with inhibitors and then stimulated with ligands or infected with bacterial pathogens for the indicated times, as previously described^42^. Cells were lysed for RNA and protein analysis.

### Bacterial infection of mice

The bacterial strains *F. novicida* U112, *Listeria monocytogenes*, and *Salmonella* typhimurium were cultured as previously described^42,70,71^. Eight- to ten-week-old, sex-matched Lyz2-Cre-expressing *Ddx6*^*fl/fl*^ and *Ddx6*^*+/+*^ littermates were infected intraperitoneally with *Listeria monocytogenes*. Body weights and clinical signs were monitored daily; the mice were euthanized on the indicated days post-infection, and the livers, spleens, and brains were harvested for CFU enumeration and analysis.

### Immunoblot analysis and antibodies

Proteins were resolved by 12.5% SDS-PAGE and transferred to PVDF membranes. After blocking, the membranes were probed with the following primary antibodies: anti-ASC (AdipoGen AG-25B-0006), anti-V5 (CST 13202), anti-caspase-1 (AdipoGen AG-20B-0042), anti-DDX6 (Bethyl A300-460A), anti-GSDMD (Abcam ab219800), anti-NLRP3 (AdipoGen AG-20B-0014), anti-AIM2 (CST 63660S), anti-EDC4 (Proteintech 17737-1-AP), anti-G3BP1 (Proteintech 66486-1-Ig), anti-NEK7 (Santa Cruz sc-393539), anti-FLAG (Sigma F3165), and anti-GAPDH (CST 2118S). HRP-conjugated anti-rabbit (CST 7074), anti-mouse (CST 7076), Mouse TrueBlot ULTRA (Rockland, 18-8817-33), and Rabbit TrueBlot ULTRA (Rockland, 18-8816-33) antibodies served as secondary antibodies.

### Immunofluorescence staining and microscopy

BMDMs were fixed (4% PFA, 15 min, RT), washed with PBS, and blocked (1× ELISA buffer + 0.1% saponin, 1 h). Primary antibodies (1:200–1:500) against the following antibodies were applied overnight at 4 °C: anti-ASC (AdipoGen AG-25B-0006; Millipore 04-147), anti-NLRP3 (AdipoGen AG-20B-0014), anti-AIM2 (CST 63660S), anti-DDX6 (Bethyl A300-460A), anti-Ataxin-2L (Proteintech 24822-1-AP), anti-NEK7 (CST 87795), anti-G3BP1 (CST 45656S; Proteintech 66486-1-Ig), and anti-EDC4 (Santa Cruz sc-376382; Proteintech 11737-1-AP). After being washed, the cells were incubated with Alexa Fluor™ 488-conjugated goat anti-rabbit (Invitrogen A-32731), Alexa Fluor™ 555-conjugated goat anti-mouse (Invitrogen A-21422), Alexa Fluor™ 488-conjugated goat anti-mouse (Invitrogen A-10680), or Alexa Fluor™ Plus 594-conjugated goat anti-rabbit (Invitrogen A-32740) secondary antibodies (1:300, 60 min, 37 °C), mounted (Vector Laboratories H-1200), and imaged on a Zeiss-LSM900 confocal microscope. Images were analyzed with ZEN black 2.3 SP1, ZEN blue 2.6, or Imaris software.

### Live-cell imaging for cell death

BMDMs (1.0 × 10^6^ cells/well) were seeded in 12-well plates and stimulated with LPS (500 ng/mL) and ATP (5 mM) or nigericin (20 μM), transfected with dsDNA (1.5 μg), or infected with *Listeria monocytogenes* (MOI = 50) or *Salmonella enterica* Typhimurium (MOI = 3). SYTOX™ Green (Invitrogen, S7020) and Hoechst (Beyotime Biotechnology, C1029) were added according to the manufacturer’s instructions. Plates were transferred to an EVOS M7000 Imaging System (Invitrogen) and maintained at 37 °C with 5% CO_2_. Images (8–16 fields/well) were captured every 5–15 min starting at time zero. Hoechst was used to label total nuclei, and SYTOX™ Green was used to label dead cells. Image analysis, masking, and quantification of dead cells were performed with Celleste 6 Image Analysis Software.

### IP-MS analysis

WT and *Asc*^*–/–*^ BMDMs were treated with LPS plus ATP or infected with *F. novicida* and were subsequently lysed in IP buffer. The anti-ASC immunoprecipitate was digested and analyzed by mass spectrometry. The MS experiment and data processing were performed by Novogene Company. Proteins detected in WT but not in *Asc*^*–/–*^ BMDMs were designated ASC-interacting proteins and are listed in Supplementary Table S1. Proteomics data are deposited in ProteomeXchange under accessions PXD054933 and PXD052497.

### Plasmid construction and co-IP experiments

The full-length sequences of DDX6 and ASC were amplified from a mouse cDNA library and subcloned and inserted into the pCDH and pcDNA3.1 vectors. Truncated DDX6 and ASC DNA sequences were amplified from the full-length cDNA plasmids and similarly subcloned and inserted into the pCDH and pcDNA3.1 vectors. Site-directed mutations (DDX6^E247Q^, DDX6^R386E^, and DDX6^G34R^) were generated using QuikChange site-directed mutagenesis kits. GFP-fused DDX6 and mCherry-fused ASC were subcloned and inserted into the pCDH vector. The DDX6 domains (IDR, Domain 1, and Domain 2) were subcloned and inserted into the Cry2-mCherry vector. For protein expression and purification, GFP-DDX6, GFP-DDX6^E247Q^, GFP-DDX6^R386E^, mCherry-ASC^PYD^, and mCherry-ASC^CARD^ were subcloned and inserted into the pET28a vector. All the plasmids were verified by DNA sequencing. The sequences of the primers used for vector construction are listed in Supplementary Table S2. For transient transfection of plasmids into HEK293T cells, Lipofectamine 3000 reagent (Invitrogen, Thermo Fisher Scientific) was used.

For IP, whole HEK293T cells collected 36 h after transfection or BMDMs (treated/untreated) were lysed in IP buffer composed of 50 mM Tris-HCl (pH 7.4), 2 mM EDTA, 150 mM NaCl, 1% NP-40, 10% glycerol, 1 mM DTT, and protease/phosphatase inhibitor cocktails (BioTools). After centrifugation, the supernatants were collected and incubated with protein A/G Plus–Agarose (Santa Cruz Biotechnology, sc-2003) or Pierce Protein A/G magnetic beads (Thermo scientific, 88802) plus 3 μg of the corresponding antibody anti-V5 (CST, 13202) or anti-FLAG beads (Sigma, A2220) for 12 h at 4 °C and then washed five times with IP buffer. Anti-ASC (AdipoGen, AG-25B-0006) and anti-DDX6 (Bethyl, A300-460A) antibodies were used for endogenous co-IP. Immunoprecipitates were eluted by boiling in SDS loading buffer for 10 min. For immunoblot analysis, the samples were resolved by SDS–PAGE, transferred to PVDF membranes, and probed with specific antibodies.

### Lentivirus production and infection

Lentiviral particles were produced by transfecting HEK293T cells with WT or mutant DDX6 plasmids plus packaging vectors. Twelve hours later, the medium was replaced with fresh complete DMEM. Viral supernatants were collected at 48 and 72 h post transfection and filtered through a 0.45 μm syringe filter. Lyz2-Cre-expressing *Ddx6*^*fl/fl*^ and *Asc*^*–/–*^ BMDMs were infected three times with the viral supernatant in the presence of 8 μg/mL polybrene as previously described^22^. Transduced cells were expanded in fresh medium for subsequent assays.

### Recombinant protein expression and purification

The GFP-DDX6, GFP-DDX6^E247Q^, GFP-DDX6^R386E^, mCherry-ASC^PYD^, and mCherry-ASC^CARD^ fusion constructs were transformed into the *Escherichia coli* strain Rosetta (DE3) pLysS for protein expression. Protein production was induced with 0.3 mM IPTG for 20 h at 18 °C. Cells were then harvested by centrifugation and resuspended in lysis buffer (50 mM Tris-HCl, 100 mM NaCl, 1 mM EDTA, 1 mM DTT and protease inhibitor) before sonication. After sonication, the supernatant was incubated with pre-equilibrated Ni-NTA affinity resin in a plastic column for 4 h at 4 °C. The resin was subsequently washed with 20 column volumes of buffer containing 50 mM imidazole, and the proteins were eluted with 5 column volumes of buffer containing 500 mM imidazole for downstream processing and experiments.

### In vitro phase separation assays

For the in vitro phase separation assays of GFP-DDX6, mCherry-ASC^PYD^, and mCherry-ASC^CARD^, the purified recombinant proteins were stored in phase separation buffer (40 mM Tris, 25 mM NaCl, 1 mM DTT, pH 7.4). To optimize the conditions for in vitro phase separation, the concentrations of recombinant protein and/or RNA in the buffer were varied as indicated. The final protein concentration was adjusted to 20 μM. RNA was added to a final concentration of 60 ng/μL, followed by the addition of 5% PEG8000 to induce phase separation. Subsequently, 20 μL of the protein mixture was loaded onto a confocal dish and imaged using an LSM 900 confocal microscope system (Zeiss).

### FRAP assays

In vitro and cellular fluorescence recovery after photobleaching (FRAP) experiments were performed on a Zeiss LSM 900 confocal microscope platform. For FRAP of the recombinant proteins, 20 μL of protein solution in ~20 μM droplets was fully or partially photobleached using 488- and 555-nm lasers at 50% laser power for 2 s. Time-lapse images were then acquired within 4–5 min after photobleaching at 10-s intervals. For FRAP of intracellular fluorescence, the puncta of GFP-DDX6 and mCherry-ASC in transfected HEK293T cells cultured in a live-cell imaging chamber at 37 °C were fully or partially photobleached with 50% laser power for 1 s using 488- and 555-nm lasers. Time-lapse images were then acquired within 1 min after photobleaching at 5-s intervals. The fluorescence intensities of the target regions were corrected by unbleached control regions and normalized to the pre-bleach intensities of the target regions.

### PolyU labeling

PolyU labeling was performed as previously described^72^. PolyU (Sigma‒Aldrich, 27416-86-0) was dissolved in 100 mM sodium acetate, pH 4.5, containing 2.5 mM sodium periodate and incubated on ice for 50 min. Activated polyU was precipitated with isopropanol, resuspended in 100 mM sodium acetate, pH 5.5, and mixed with Alexa Fluor™ 594 Hydrazide (Thermo Fisher Scientific, A20502) at a 1:2 molar ratio (polyU:dye). The reaction proceeded at 4 °C for 24 h. Labeled polyU was precipitated, washed extensively with isopropanol, and reconstituted in DEPC-treated water to the desired concentration.

### Analysis of bulk and single-cell RNA-seq data

Bulk RNA-seq data for PBMCs and monocytes from 284 healthy donors and 125 patients with sepsis were downloaded from GEO (GSE205672)^25^. *DDX6* expression in each cell population was compared between groups and visualized with violin plots. Single-cell RNA-seq data from the BALF of 7 patients with COVID-19 (3 mild/moderate, 4 severe/critical) were obtained from the GEO (GSE158055)^26^. Raw counts were processed in Seurat v4.1.0^73^, followed by normalization, variable-gene selection (top 2000), scaling, and PCA; batch effects were removed using Harmony^74^. The top 30 PCs were used for neighbor finding (FindNeighbors) and clustering (FindClusters, resolution 0.5) to yield 15 clusters. The top 6 principal components were selected for *t*-SNE visualization. The cell types were assigned using canonical markers. *DDX6* expression was extracted (FetchData) and compared between the mild and severe groups using a two-sided Wilcoxon test with Benjamini–Hochberg correction. Violin plots were generated with ggplot2 and ggpubr.

### Flow cytometry analysis

For flow cytometric analysis of BMDMs (CD11b^+^F4/80^+^) and alveolar macrophages (CD11c^+^F4/80^+^), cell preparation and staining with cell surface markers were carried out as described previously^22,71^, and the cells were incubated with anti-CD11b (BioLegend, 101212), anti-CD11c (BioLegend, 117322) and anti-F4/80 (BioLegend, 123108) antibodies and then analyzed on a BD LSRFortessa Cell Analyzer (BD Biosciences).

### SEM

WT BMDMs were seeded onto glass slides at a density of 1 × 10⁵ cells per well and cultured overnight. The cells were pretreated with glycine (5 mM) and then stimulated with LPS (500 ng/mL, 4 h) and nigericin (20 μM, 45 min) to activate the NLRP3 inflammasome. After stimulation, the samples were fixed in electron microscopy fixative solution (Servicebio, G1102-1.5 ML) and imaged using a Hitachi SU-8010 scanning electron microscope at the Imaging Core Facility of Shandong University.

### Preparation of tissue samples for HE staining

The superior lobes of the right lung, liver, and brain were fixed in 10% formalin, and 5 μm sections were stained with H&E and examined under a microscope.

### ELISA

In vivo and in vitro samples were analyzed for cytokine release using ELISA MAX Standard Sets from BioLegend (Mouse IL-1β, 432601; Mouse IL-6, 431301; Mouse TNF, 430901) according to the manufacturer’s instructions.

### Statistical analyses

The data are presented as the mean ± standard error of the mean (SEM). Statistical analyses were performed using two-way ANOVA, Wilcoxon signed-rank tests, and 2-tailed Student’s *t*-test and log-rank test. *P* values of 0.05 or less were considered significant.

## Supplementary information

Supplementary Information

Supplementary Table S1

Supplementary Table S2
