# Supplementary material for: RNA helicase DDX6 governs ASC speck formation in P-bodies and the transition to stress granules via phase separation during inflammasome activation
Source: Cell Discov. 2026 Jun 24;12:44. doi: 10.1038/s41421-026-00898-1 (PMC13294372; doi:10.1038/s41421-026-00898-1)
Supplement: Supplementary file 1 — Supplementary Information [file 41421_2026_898_MOESM1_ESM.pdf]

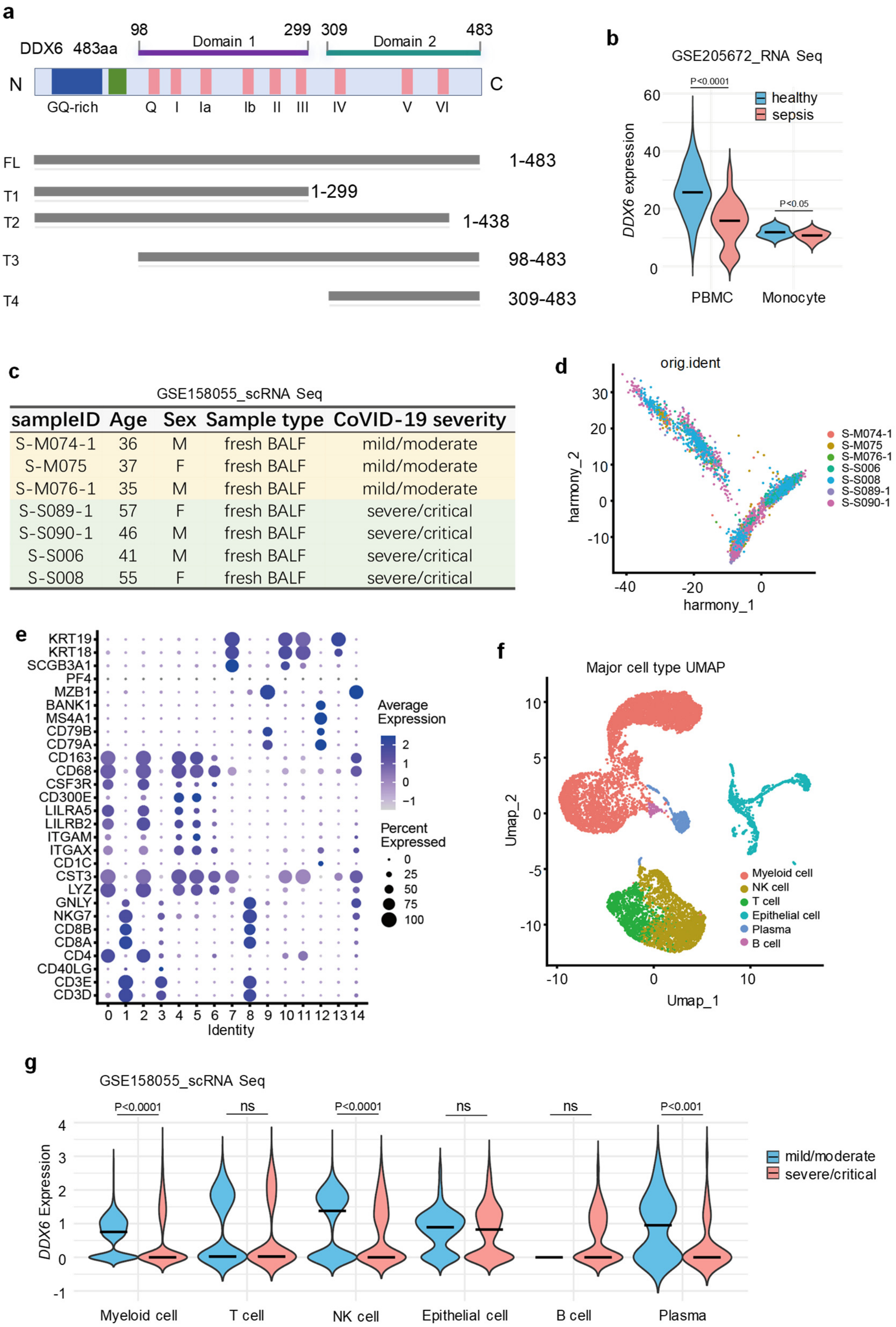

GSE158055_scRNA Seq

sampleID	Age	Sex	Sample type	CoVID-19 severity
S-M074-1	36	M	fresh BALF	mild/moderate
S-M075	37	F	fresh BALF	mild/moderate
S-M076-1	35	M	fresh BALF	mild/moderate
S-S089-1	57	F	fresh BALF	severe/critical
S-S090-1	46	M	fresh BALF	severe/critical
S-S006	41	M	fresh BALF	severe/critical
S-S008	55	F	fresh BALF	severe/critical

Supplementary Figure S1. Single-cell RNA sequencing analysis of DDX6 expression.

a, Schematic representation of full-length and truncated DDX6 plasmids T1-T4.
b, Gene expression analysis of *DDX6* in peripheral blood mononuclear cells (PBMCs) and monocytes from RNA sequencing data (GSE205672) of 284 healthy subjects and 125 sepsis patients.
c, Summary of sample information and patient demographics for single-cell RNA sequencing analysis.
d, Batch correction results of single-cell RNA sequencing data (**b**) using Harmony.
e, Dot plot showing the expression of the selected marker genes for each cluster.
f, Uniform manifold approximation and projection (UMAP) plot illustrating the distribution of immune and epithelial cells colored by cluster in (**e**). Myeloid cells were characterized by high expression of *LILRB2*, *LILRA5*, *CD300E*, *LYZ*, *CST3*, *CD68*, *ITGAM*, *ITGAX*, and *CD163* (clusters 0, 2, 4, 5, 6, and 7); T cells by *CD3D*, *CD3E*, *CD4*, and *CD8A* (cluster 3); NK cells by *NKG7* (clusters 1 and 8); B cells by *CD79A*, *CD79B*, *CD19*, and *BANK1* (cluster 12); Epithelial cells by *SCGB3A1*, *KRT18*, and *KRT19* (clusters 7, 10, 11, and 13); Plasma cells by *MZB1* (clusters 9 and 14).
g, Gene expression analysis of *DDX6* in myeloid cells, T cells, NK cells, epithelial cells, B cells, and plasma from single-cell RNA sequencing data (GSE158055) of bronchoalveolar lavage fluid (BALF) from 3 mild and 4 severe COVID-19 patients.
Data represent Mean±SEM for (**b**, **g**), 2-sided Wilcoxon rank-sum tests, *p* value is indicated in the graph.

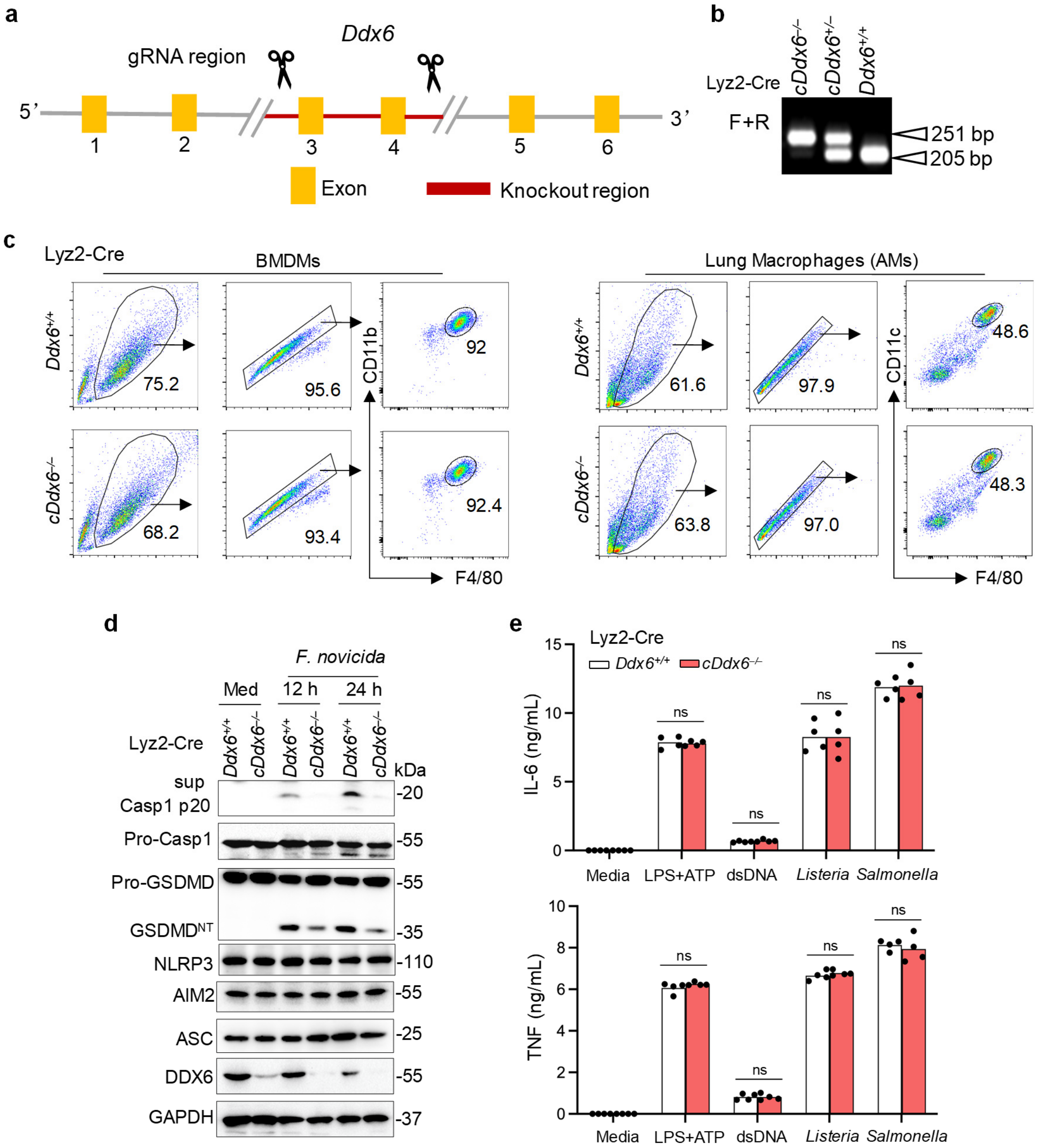

Supplementary Figure S2. Characterization of macrophage differentiation in the absence of DDX6.

a, Targeting strategy for generating *Ddx6*$^{fl/fl}$ mice using CRISPR/Cas9. Yellow boxes represent exons of the *Ddx6* gene; the red line indicates the floxed region encompassing exons 3 and 4 targeted for deletion.

b, Genotyping of offspring from heterozygous *Ddx6*$^{fl/fl}$ mouse crosses. A 251 bp PCR product corresponds to the floxed allele, while a 205 bp product corresponds to the wild-type allele.

c, Representative flow cytometry plots of BMDMs (CD11b^{+}F4/80^{+}) and lung alveolar macrophages (AMs, CD11c^{+}F4/80^{+}) derived from Lyz2-Cre expressing *Ddx6*$^{fl/fl}$ (*cDdx6*$^{-/-}$) and *Ddx6*$^{+/+}$ mice.

d, Immunoblot analysis of pro-caspase-1 (Pro-Casp1), its subunit p20, DDX6, NLRP3, AIM2, ASC, full-length and cleaved GSDMD ($GSDMD^{NT}$) in Lyz2-Cre expressing *Ddx6*$^{fl/fl}$ (*cDdx6*$^{-/-}$) and *Ddx6*$^{+/+}$ BMDMs without treatment (Med) or infected with *F. novicida* (200 MOI, 12 h and 24 h) for AIM2 inflammasome activation.

e, Analysis of IL-6 and TNF in WT and *Ddx6* deficient BMDMs without treatment (Media) or stimulated with LPS (500 ng/mL, 4 h) and ATP (5 mM, 60 min), transfected with dsDNA (1.5 μg, 2 h), infected with *Listeria monocytogenes* (50 MOI, 6 h), and *Salmonella enterica* Typhimurium (3 MOI, 4 h) (n=4 biologically independent samples).

Data are from 3 (**e**) or representative of 3 independent experiments with similar results (**b**-**d**). Data represent Mean±SEM for (**e**), 2-sided Student's t test without multiple-comparisons correction.

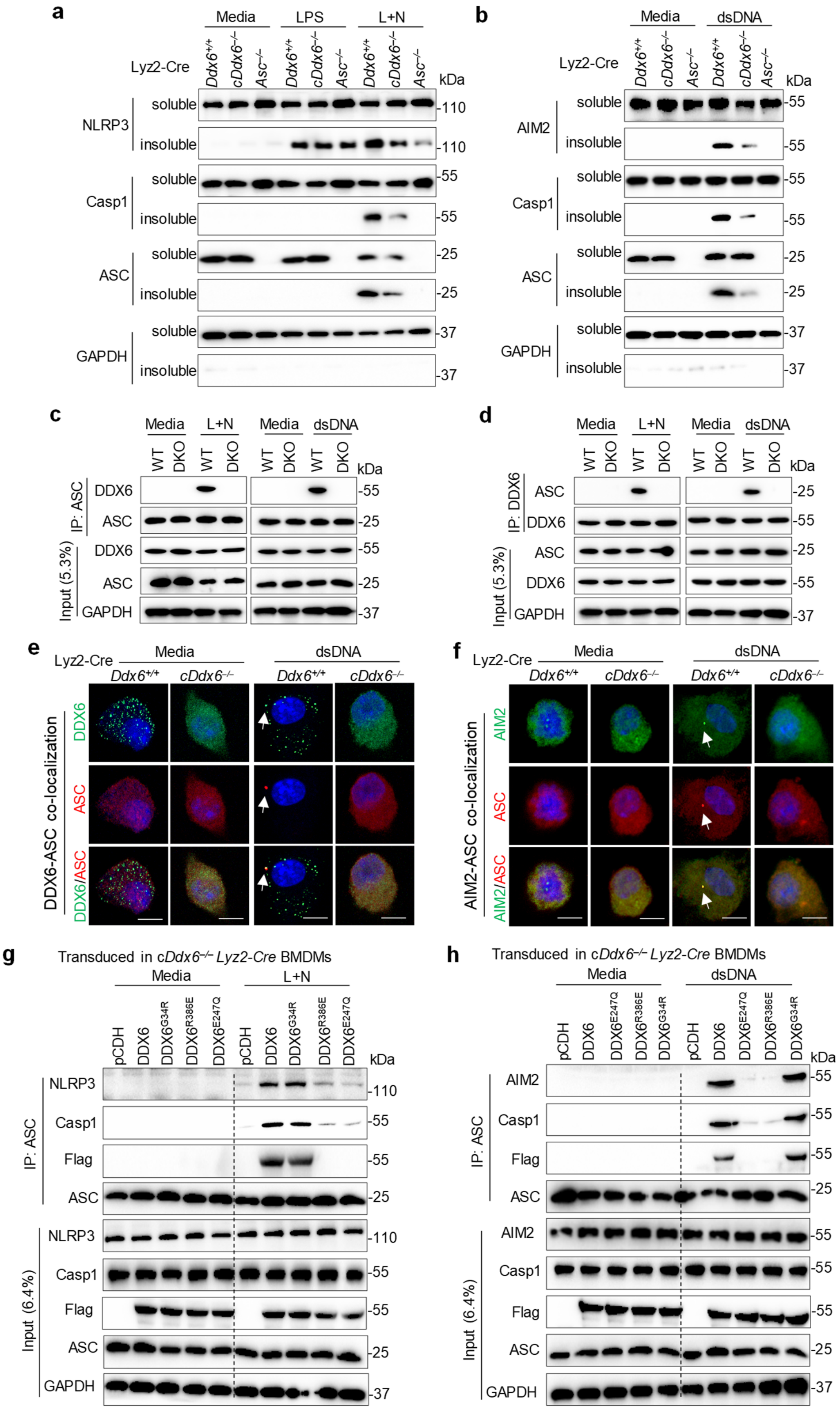

Supplementary Figure S3. DDX6 promotes ASC speck formation.

a, Immunoblot analysis of ASC, NLRP3, caspase-1, and GAPDH in the insoluble pellet and soluble lysaste separated by centrifugation with 12,000 g for 10 min of Lyz2-Cre expressing *Ddx6*$^{fl/fl}$ (*cDdx6*$^{-/-}$) and *Ddx6*$^{+/+}$ BMDMs, and *Asc*$^{-/-}$ BMDMs. Cells were untreated (Media), stimulated with LPS (500 ng/mL, 4 h), or LPS (500 ng/mL, 4 h) plus nigericin (20 μM, 60 min) for NLRP3 inflammasome activation.
b, Immunoblot analysis of ASC, AIM2, caspase-1, and GAPDH in the insoluble pellet and soluble lysaste of Lyz2-Cre expressing *Ddx6*$^{fl/fl}$ (*cDdx6*$^{-/-}$) and *Ddx6*$^{+/+}$ BMDMs, and *Asc*$^{-/-}$ BMDMs without treatment (Media), transfected with dsDNA (1.5 μg, 2 h) for AIM2 inflammasome activation.
c,**d** Endogenous Co-IP analysis of DDX6 with anti-ASC immunoprecipitates (**c**) and of ASC with anti-DDX6 immunoprecipitates (**d**) in WT and *Nlrp3*$^{-/-}$*Aim2*$^{-/-}$ (DKO) BMDMs. Cells were treated with the NLRP3 activator LPS plus nigericin (LPS, 500 ng/mL, 4 h and nigericin, 20 μM, 20 min) or transfected with the AIM2 activator dsDNA (1.5 μg, 1 h).
e,**f** Confocal microscopy analysis of co-localization of DDX6 and ASC (**e**), and ASC and AIM2 (**f**) in Lyz2-Cre expressing *Ddx6*$^{fl/fl}$ (*cDdx6*$^{-/-}$) and *Ddx6*$^{+/+}$ BMDMs transfected with dsDNA (1.5 μg, 2 h) for AIM2 inflammasome activation. Arrows indicate co-localized puncta. Scale bars: 10 μm.
g, Co-IP analysis endogenous ASC interactions with DDX6, caspase-1, and NLRP3 in Lyz2-Cre expressing *Ddx6*$^{fl/fl}$ (*cDdx6*$^{-/-}$) BMDMs transduced with WT DDX6 and point mutations as indicated, without treatment (Media) or further stimulated with LPS plus nigericin (LPS, 500 ng/mL, 4 h and nigericin, 20 μM, 20 min) for NLRP3 inflammasome activation.
h, Co-IP analysis endogenous ASC interactions with DDX6, caspase-1, and AIM2 in Lyz2-Cre expressing *Ddx6*$^{fl/fl}$ (*cDdx6*$^{-/-}$) BMDMs transduced with WT DDX6 and point mutations as indicated, without treatment (Media) or further transfected with dsDNA (1.5 μg, 60 min) for AIM2 inflammasome activation.
Data are representative of 3 (**a**-**g**) or 2 (**h**) independent experiments with similar results.

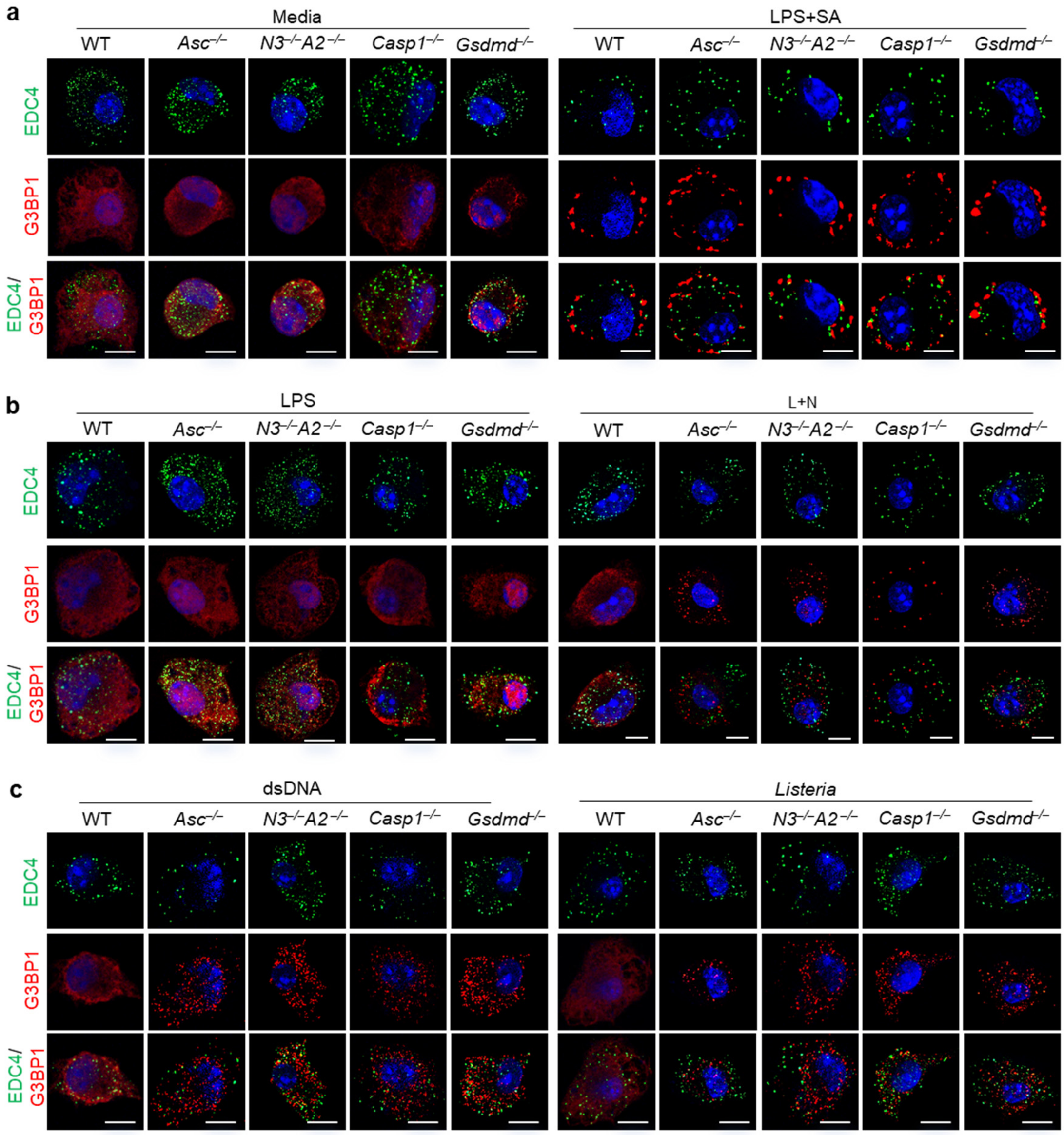

Supplementary Figure S4. Subcellular localization of EDC4 and G3BP1 in BMDMs in response to diverse stimuli.

a, Confocal microscopy analysis of EDC4 and G3BP1 co-localization in WT, *Nlrp3*$^{-/-}$*Aim2*$^{-/-}$ (*N3*$^{-/-}$*A2*$^{-/-}$), *Asc*$^{-/-}$, *Caspase-1*$^{-/-}$ (*Casp1*$^{-/-}$), and *Gsdmd*$^{-/-}$ BMDMs without treatment (Media) or stimulated with LPS plus sodium arsenite (LPS, 500 ng/mL, 4 h and SA, 50 μM, 30 min). Scale bars: 10 μm.

b, Confocal microscopy analysis of EDC4 and G3BP1 co-localization in WT, *Nlrp3*$^{-/-}$*Aim2*$^{-/-}$ (*N3*$^{-/-}$*A2*$^{-/-}$), *Asc*$^{-/-}$, *Caspase-1*$^{-/-}$ (*Casp1*$^{-/-}$), and *Gsdmd*$^{-/-}$ BMDMs stimulated with LPS alone (LPS, 500 ng/mL, 4 h) or LPS plus nigericin (LPS, 500 ng/mL, 4 h and nigericin, 20 μM, 30 min). Scale bars: 10 μm.

c, Confocal microscopy analysis of EDC4 and G3BP1 co-localization in WT, *Nlrp3*$^{-/-}$*Aim2*$^{-/-}$ (*N3*$^{-/-}$*A2*$^{-/-}$), *Asc*$^{-/-}$, *Caspase-1*$^{-/-}$ (*Casp1*$^{-/-}$), and *Gsdmd*$^{-/-}$ BMDMs

transfected with dsDNA (1.5 μg, 2 h) or infected with *Listeria monocytogenes* (50 MOI, 2 h). Scale bars: 10 μm.
Data are representative of 3 independent experiments with similar results.

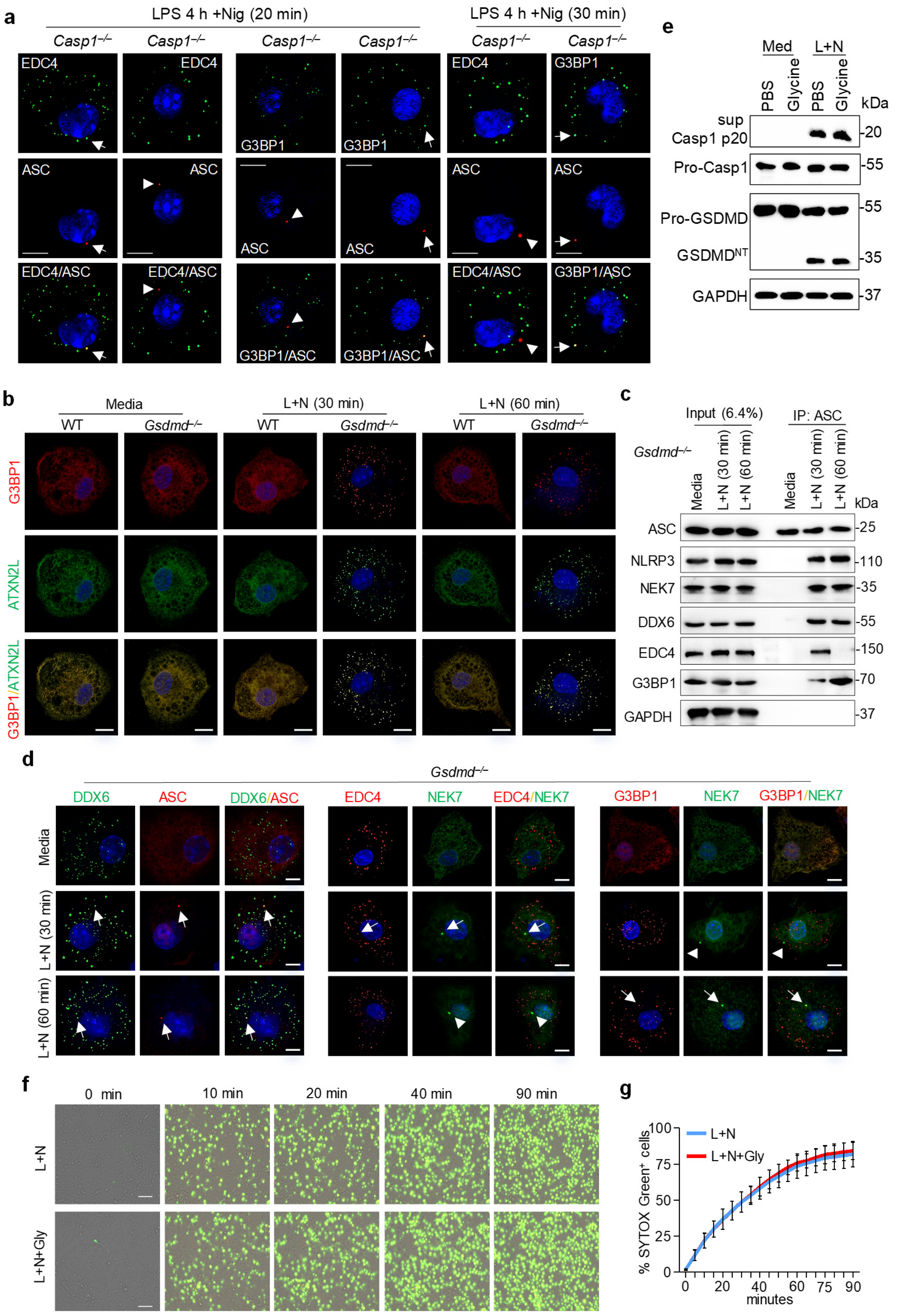

Supplementary Figure S5. NLRP3 and NEK7 co-localize with ASC-DDX6 in PBs and SGs.

a, Confocal microscopy analysis of ASC co-localization with EDC4 or G3BP1 in *Caspase-1*$^{-/-}$ (*Casp1*$^{-/-}$) BMDMs stimulated with LPS plus nigericin (LPS, 500 ng/mL, 4 h and nigericin, 20 μM, 20 min and 30 min) for NLRP3 inflammasome activation. Arrows indicate the co-localized puncta, and arrowheads indicate the non-colocalized puncta. Scale bars: 10 μm.
b, Confocal microscopy analysis of G3BP1 and ATXN2L in WT and *Gsdmd*$^{-/-}$ BMDMs. Cells were untreated (Media) or stimulated with LPS plus nigericin (LPS, 500 ng/mL, 4 h and nigericin, 20 μM, 30 min and 60 min). Scale bars: 10 μm.
c, Endogenous Co-IP analysis of ASC interactions with NLRP3, NEK7, DDX6, EDC4, and G3BP1 in *Gsdmd*$^{-/-}$ BMDMs. Cells were untreated (Media) or stimulated with LPS plus nigericin (LPS, 500 ng/mL, 4 h and nigericin, 20 μM, 30 min and 60 min).
d, Co-localization analysis of DDX6 with ASC, NEK7 with EDC4, and NEK7 with G3BP1 in *Gsdmd*$^{-/-}$ BMDMs. Cells were untreated (Media) or stimulated with LPS plus nigericin (LPS, 500 ng/mL, 4 h and nigericin, 20 μM, 30 min and 60 min). Arrows indicate the co-localized puncta, and arrowheads indicate the non-colocalized puncta. Scale bars: 10 μm.
e, Immunoblot analysis of pro-caspase-1 (Pro-Casp1), its subunit p20, full-length and cleaved GSDMD ($GSDMD^{NT}$) in WT BMDMs pretreated with glycine (5 mM) and stimulated with LPS plus nigericin (LPS, 500 ng/mL, 4 h and nigericin, 20 μM, 30 min) for NLRP3 inflammasome activation.
f, Representative images of SYTOX Green positive cells in untreated and glycine-pretreated (5 mM) WT BMDMs and further stimulated with LPS plus nigericin (LPS, 500 ng/mL, 4 h and nigericin, 20 μM) for indicated times. Scale bars: 50 μm.
g, Real-time quantitative live-cell imaging and analysis of cell death in untreated and glycine-pretreated (5 mM) WT BMDMs and further stimulated with LPS plus nigericin (LPS, 500 ng/mL, 4 h and nigericin, 20 μM) for indicated times. (n=8 random fields; 3 independent experiments).
Data are representative of 3 independent experiments with similar results (**a-g**).

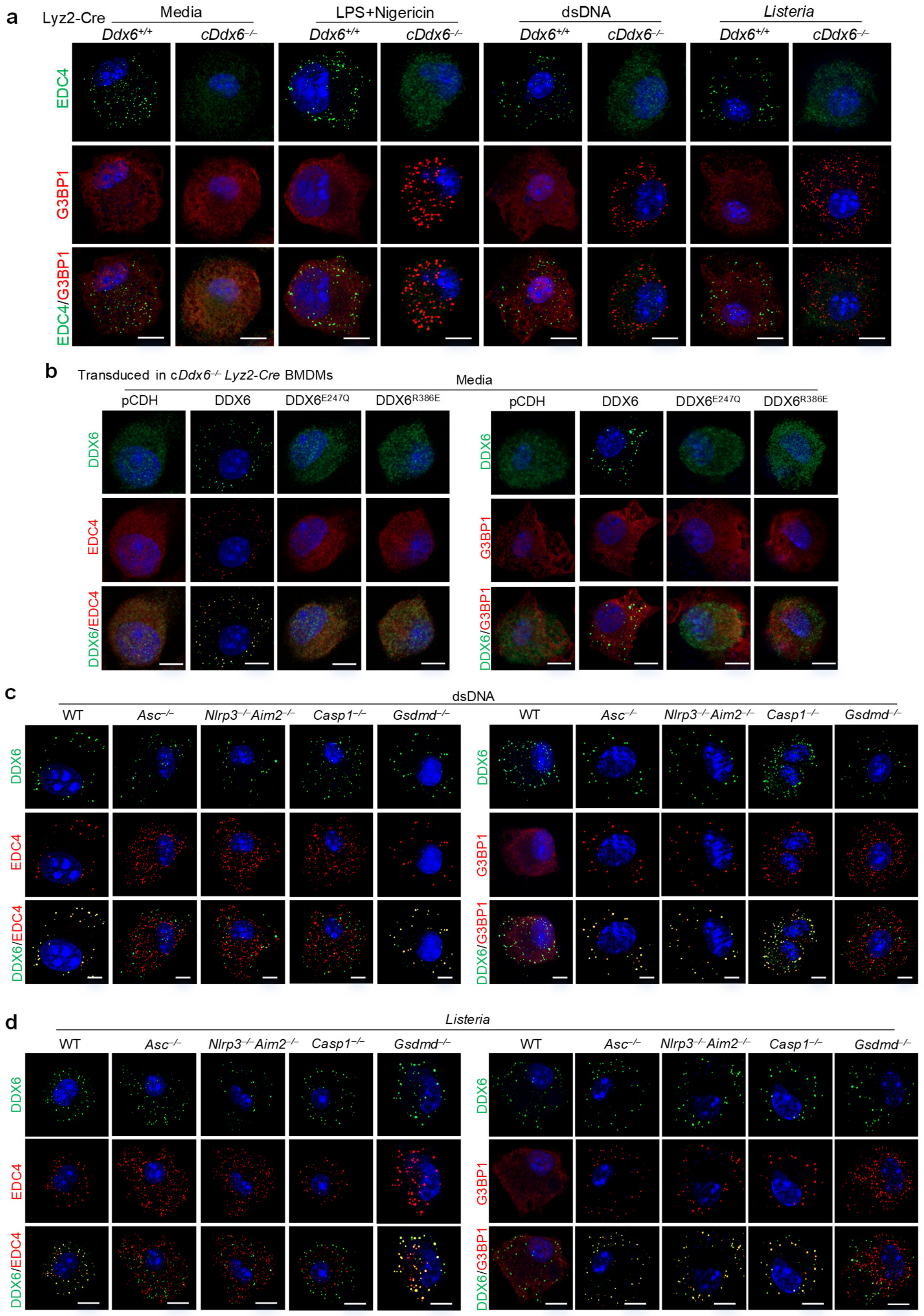

Supplementary Figure S6. DDX6 deficiency inhibits PB formation and induces SG formation in response to inflammasome activation.

a, Confocal microscopy analysis of EDC4 and G3BP1 co-localization in Lyz2-Cre expressing *Ddx6*$^{fl/fl}$ (*cDdx6*$^{–/–}$) and *Ddx6*$^{+/+}$ BMDMs without treatment (Media) or stimulated with LPS (500 ng/mL, 4 h) and nigericin (20 μM, 30 min), transfected with dsDNA (1.5 μg, 2 h), infected with *Listeria monocytogenes* (50 MOI, 2 h). Scale bars: 10 μm.
b, Confocal microscopy analysis of DDX6 co-localization with EDC4 or G3BP1 in Lyz2-Cre expressing *Ddx6*$^{fl/fl}$ (*cDdx6*$^{–/–}$) BMDMs transduced with WT DDX6 and mutants as indicated. Scale bars: 10 μm.
c, Confocal microscopy analysis of DDX6 co-localization with EDC4 or G3BP1 in WT, *Nlrp3*$^{–/–}$*Aim2*$^{–/–}$, *Asc*$^{–/–}$, *Caspase-1*$^{–/–}$ (*Casp1*$^{–/–}$), and *Gsdmd*$^{–/–}$ BMDMs transfected with dsDNA (1.5 μg, 2 h) for AIM2 inflammasome activation. Scale bars: 10 μm.
d, Confocal microscopy analysis of DDX6 co-localization with EDC4 or G3BP1 in WT, *Nlrp3*$^{–/–}$*Aim2*$^{–/–}$, *Asc*$^{–/–}$, *Caspase-1*$^{–/–}$ (*Casp1*$^{–/–}$), and *Gsdmd*$^{–/–}$ BMDMs infected with *Listeria monocytogenes* (50 MOI, 2 h) for both NLRP3 and AIM2 inflammasome activation. Scale bars: 10 μm.
Data are representative of 3 (**a-d**) independent experiments with similar results.

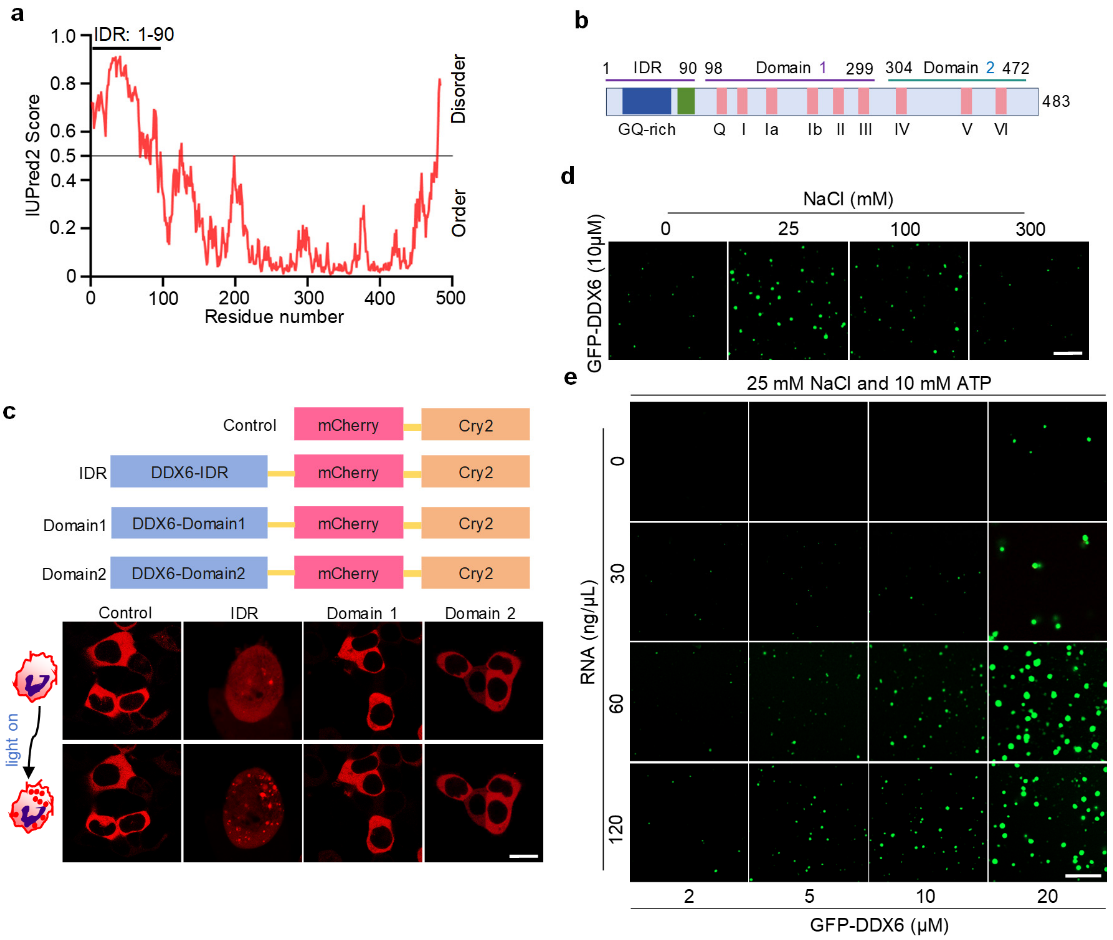

Supplementary Figure S7. Intrinsically disordered region of DDX6 facilitates LLPS of DDX6.

a, The intrinsically disordered tendency of DDX6. IUPred2 assigned scores of disordered tendencies between 0 and 1 to the sequences shown.

b, The domain structure of DDX6 including IDR (1-90 amino acid), domain 1 (98-299 amino acid) and domain 2 (304-472 amino acid).

c, Time-lapse images of HEK293T cells expressing constructs containing indicated DDX6 domains (IDR, Domain 1, and Domain 2) linked to mCherry and Cry2, before and after light activation (488 nm). mCherry–Cry2 fusion alone was used as control. Scale bar: 10 μm.

d, Representative micrographs of GFP-DDX6 droplets (10 μM) in the presence of RNA (60 ng/μL), ATP (10 mM), and different concentrations of NaCl as indicated. Scale bar: 5 μm.

e, Representative micrographs of GFP-DDX6 droplets (2, 5, 10, 20 μM) in the presence of NaCl (25 mM), and ATP (10 mM) with different concentrations of RNA as indicated. Scale bar: 5 μm.

Data are representative of 3 (**c**-**e**) independent experiments with similar results.

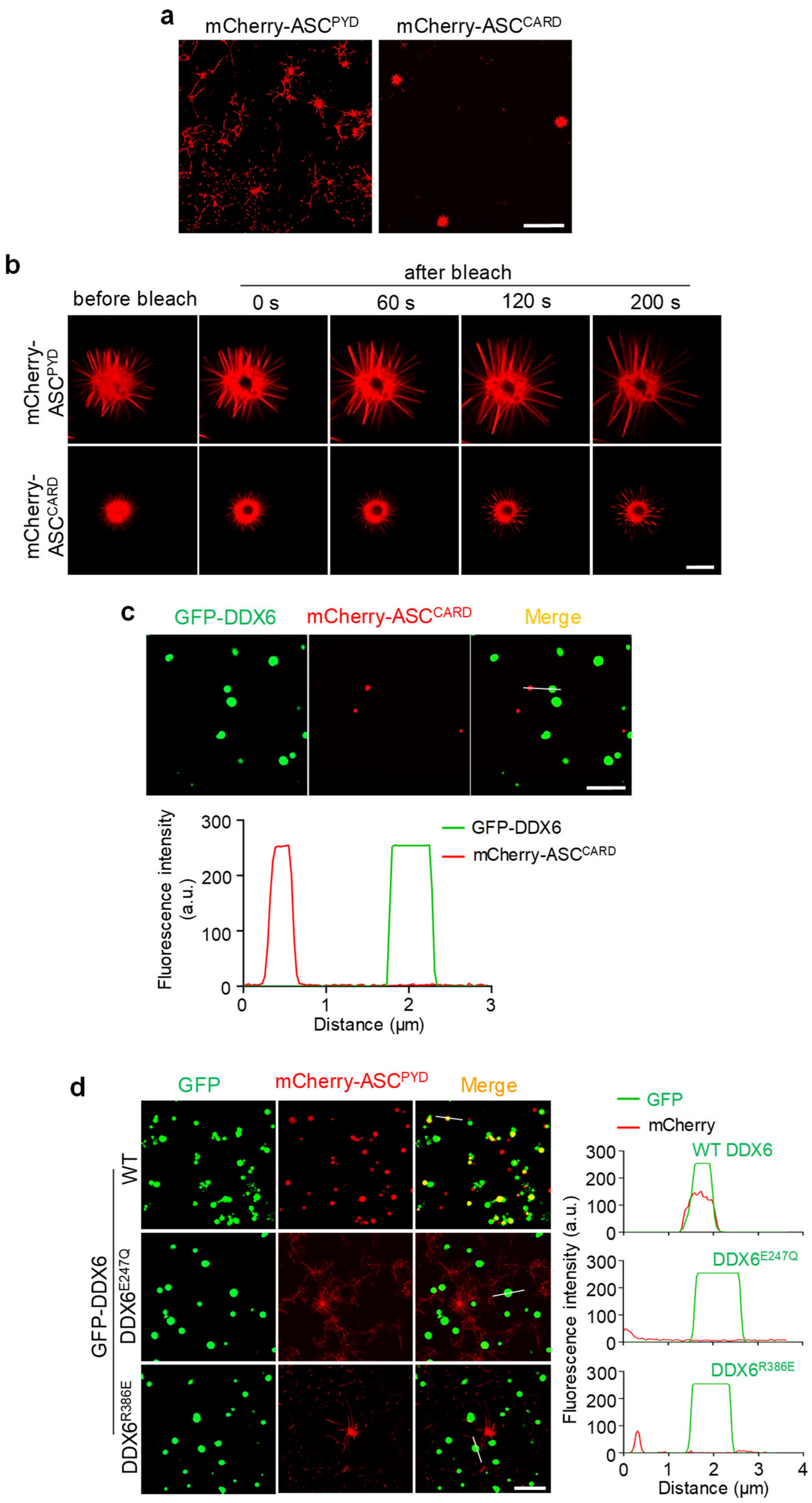

Supplementary Figure S8. WT DDX6 droplets fuse with ASC^{PYD}.

a, Representative micrographs of mCherry-ASC^{PYD} (10 μM) and mCherry-ASC^{CARD} (10 μM) aggregates. Scale bar: 5 μm.
b, Representative micrographs of mCherry-ASC^{PYD} (10 μM) and mCherry-ASC^{CARD} (10 μM) undergoing FRAP over 200 s. Scale bar: 2 μm.
c, Representative micrographs (upper) and quantitative line profile along the white line (lower) of GFP-DDX6 droplets (20 μM) and mCherry-ASC^{CARD} (20 μM) in the presence of RNA (60 ng/μL) and ATP (10 mM). Scale bar: 5 μm.
d, Representative micrographs (left) and quantitative line profile along the white line (right) of mCherry-ASC^{PYD} and GFP fused with WT DDX6 or DDX6 mutants as indicated in the presence of RNA (60 ng/μL) and ATP (10 mM). Scale bar: 5 μm.
Data are representative of 3 independent experiments with similar results (**a**-**d**).

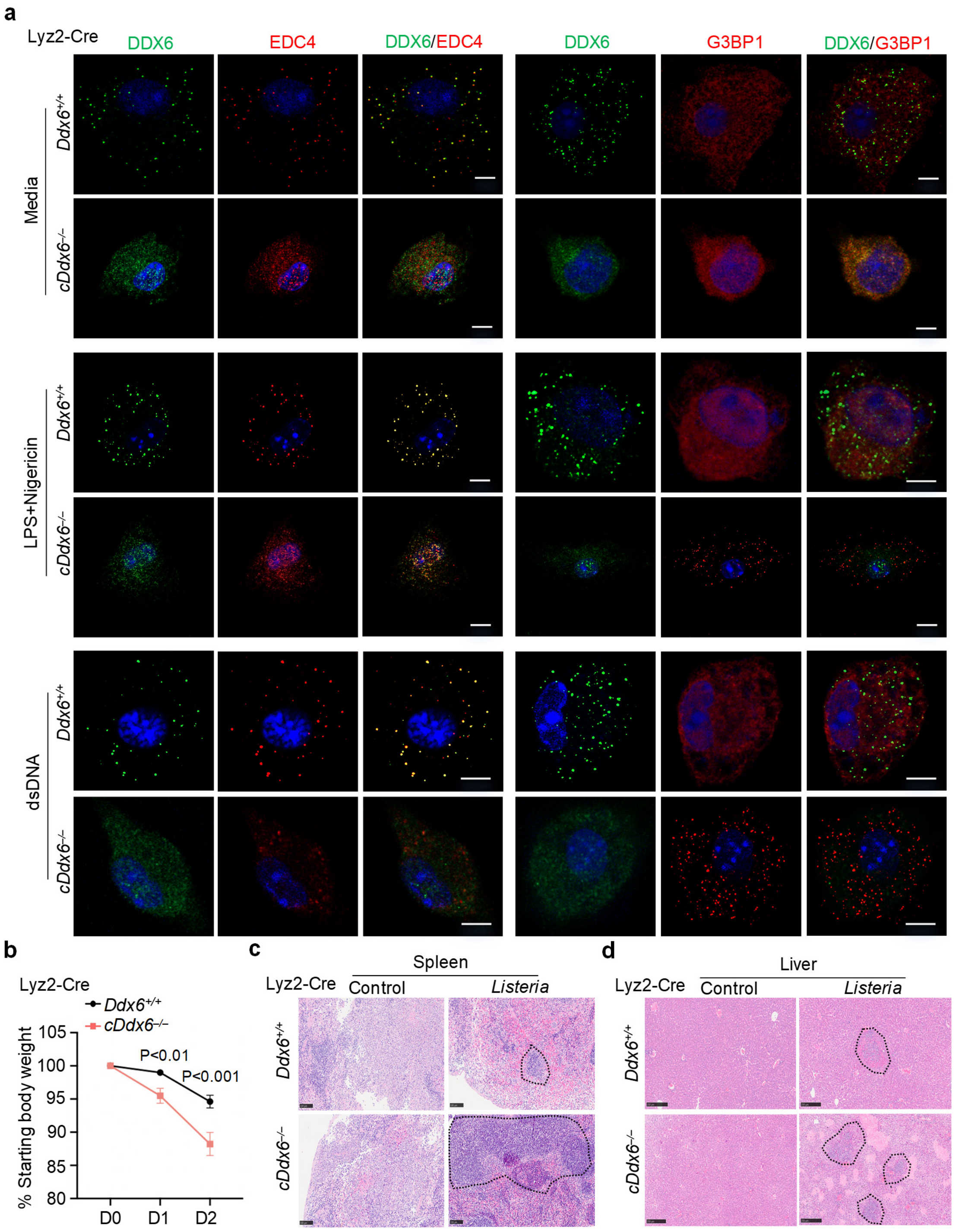

Supplementary Figure S9. Characterization of DDX6 in host defense against *Listeria monocytogenes* infection.

a, Confocal microscopy analysis of DDX6 co-localization with EDC4 or G3BP1 in Lyz2-Cre expressing *Ddx6*$^{fl/fl}$ (*cDdx6*$^{-/-}$) and *Ddx6*$^{+/+}$ BMDMs without treatment

(Media) or stimulated with LPS (500 ng/mL, 4 h) and nigericin (20 μM, 30 min), and transfected with dsDNA (1.5 μg, 2 h). Scale bars: 10 μm.
b, Lyz2-Cre expressing *Ddx6*$^{fl/fl}$ (*cDdx6*$^{-/-}$, n=6) and littermate control *Ddx6*$^{+/+}$ (n=6) female mice were intraperitoneally infected with *Listeria monocytogenes* (6.0x10^5 CFUs per mouse), and body weight were monitored.
c,**d**, H&E staining of spleen (**c**) and liver (**d**) sections from uninfected and *Listeria monocytogenes*-infected mice in (**b**). Dashed outlines indicate immune cell infiltrates. Scale bars: 100 μm.
Data are representative of 3 independent experiments with similar results (**a**-**d**). Data represent Mean±SEM for (**b**), 2-sided Student's t test without multiple-comparisons correction, *p* value is indicated in the graph.

Supplementary Table S1. List of ASC-interacting proteins and detected peptides of DDX6 and ASC by IP-MS analysis.
Supplementary Table S2. Sequence of oligos used in this manuscript.
